# A Comprehensive Review about the Molecular Structure of Severe Acute Respiratory Syndrome Coronavirus 2 (SARS-CoV-2): Insights into Natural Products against COVID-19

**DOI:** 10.3390/pharmaceutics13111759

**Published:** 2021-10-21

**Authors:** Essa M. Saied, Yousra A. El-Maradny, Alaa A. Osman, Amira M. G. Darwish, Hebatallah H. Abo Nahas, Gniewko Niedbała, Magdalena Piekutowska, Mohamed A. Abdel-Rahman, Bassem A. Balbool, Ahmed M. Abdel-Azeem

**Affiliations:** 1 Chemistry Department, Faculty of Science, Suez Canal University, Ismailia 41522, Egypt; 2 Institute for Chemistry, Humboldt Universität zu Berlin, Brook-Taylor-Str. 2, 12489 Berlin, Germany; 3 Microbiology Department, High Institute of Public Health, Alexandria University, Alexandria 21526, Egypt; hiph.ymaradny@alexu.edu.eg; 4 Department of Pharmaceutical Chemistry, Faculty of Pharmacy, New Giza University, Newgiza, km 22 Cairo-Alexandria Desert Road, Cairo 12256, Egypt; ala.awad@ngu.edu.eg; 5 Food Technology Department, Arid Lands Cultivation Research Institute (ALCRI), City of Scientific Research and Technological Applications (SRTA City), Alexandria 21934, Egypt; amiragdarwish@yahoo.com; 6 Zoology Department, Faculty of Science, Suez Canal University, Ismailia 41522, Egypt; hebatallah_hassan@science.suez.edu.eg (H.H.A.N.); mohamed_hassanain@science.suez.edu.eg (M.A.A.-R.); 7 Department of Biosystems Engineering, Faculty of Environmental and Mechanical Engineering, Poznań University of Life Sciences, Wojska Polskiego 50, 60-627 Poznań, Poland; gniewko.niedbala@up.poznan.pl; 8 Department of Geoecology and Geoinformation, Institute of Biology and Earth Sciences, Pomeranian University in Słupsk, Partyzantów 27, 76-200 Słupsk, Poland; magdalena.piekutowska@apsl.edu.pl; 9 Faculty of Biotechnology, October University for Modern Sciences and Arts, Giza 12585, Egypt; Bbalbool@msa.edu.eg; 10 Botany and Microbiology Department, Faculty of Science, Suez Canal University, Ismailia 41522, Egypt

**Keywords:** coronavirus, SARS-CoV-2, virus lifecycle, COVID-19, virus detection, molecular structure, vaccines, therapeutic approach, natural products, antivirals, antioxidants

## Abstract

In 2019, the world suffered from the emergence of COVID-19 infection, one of the most difficult pandemics in recent history. Millions of confirmed deaths from this pandemic have been reported worldwide. This disaster was caused by SARS-CoV-2, which is the last discovered member of the family of *Coronaviridae*. Various studies have shown that natural compounds have effective antiviral properties against coronaviruses by inhibiting multiple viral targets, including spike proteins and viral enzymes. This review presents the classification and a detailed explanation of the SARS-CoV-2 molecular characteristics and structure–function relationships. We present all currently available crystal structures of different SARS-CoV-2 proteins and emphasized on the crystal structure of different virus proteins and the binding modes of their ligands. This review also discusses the various therapeutic approaches for COVID-19 treatment and available vaccinations. In addition, we highlight and compare the existing data about natural compounds extracted from algae, fungi, plants, and scorpion venom that were used as antiviral agents against SARS-CoV-2 infection. Moreover, we discuss the repurposing of select approved therapeutic agents that have been used in the treatment of other viruses.

## 1. Introduction

Coronaviruses (CoVs) are single-stranded RNA viruses that can infect both animals and humans [1]. Tyrell and Bynoe were the first to investigate these viruses in 1966. The viruses were given the name “coronaviruses” because of their spherical virions, which has a shell and surface projections similar to a solar corona. The word corona is a Latin word meaning crown, and there are four subfamilies that have been identified so far: alpha, beta, gamma, and delta. The alpha and beta subfamilies originated in mammals, mainly bats, while the gamma and delta coronaviruses originated in pigs and birds [2]. By the end of 2019 in Wuhan, coronavirus made the transfer from animal to human, leading to the coronavirus disease (COVID-19). This disease has been found to be triggered by a novel coronavirus known as severe acute respiratory syndrome coronavirus 2 (SARS-CoV-2). Later, this novel coronavirus has been isolated, and its genome sequence was determined by Chinese scientists [3]. COVID-19 disease has been characterized by lower and upper respiratory tract infection and further critical complications which lead to premature mortality. Within a week, SARS-CoV-2 infected around 70,000 people and caused over 1800 deaths; therefore, the World Health Organization (WHO) declared COVID-19 disease as a global pandemic disease [4,5]. According to Worldometer, the COVID-19 disease impacted 223 nations, with over than 234 million infected people, more than 4.8 million deaths, and 211 million recovered patients [6]. In an attempt to restrict the dissemination of SARS-CoV-2, governments all over the world have instituted social distancing measures and stringent lock downs [7].

SARS-CoV-2 infection cycle starts by the binding of the spike (S) protein of SARS-CoV-2 to the host cell membrane. The S protein is cleaved by transmembrane protease serine 2 (TMPRSS2) into two subunits (S1 and S2), which play a key role in the receptor recognition and cell membrane fusion process [8]. The S1 subunit contains a receptor-binding domain (C-terminal domain) that recognizes and binds to the host angiotensin converting enzyme 2 receptor (ACE2), while the S2 subunit mediates viral cell membrane fusion and the release of the virus genome into the infected cell by forming a six-helical bundle via the two-heptad repeat domain [9,10]. The replication and transcription of the viral RNA occur in the cytoplasm to produce ensemble viruses which exit the infected cell via exocytosis to infect another cell and repeat the infection cycle again [11,12].

SARS-CoV-2 infection has been shown to induce an extreme innate immune response and to increase the level of cytokines and chemokines in bronchial, which lead to accumulation of monocytes, leukocytes, natural killer cells, and interleukins [13]. The high expression of these mediators induces massive inflammatory reactions which attenuate the efficacy of the lungs and cause cough, fever, and pneumonia-like symptoms. Recently, gastrointestinal symptoms and silent infections, particularly among young children, have been described [14,15]. It has been also reported that the S protein of the virus can bind to the ACE2 receptors which are expressed in the neuronal tissue and the cerebral capillary endothelium which lead to damage of neural and deterioration of cerebral capillary in COVID-19-infected patients [16,17,18].

To date, a huge amount of knowledge has been acquired about SARS-CoV-2 virus including its molecular structure, lifecycle, and its interactions with the host cell. Such information has led to the development of several vaccines, together with potential antiviral drugs. The Food and Drug Administration (FDA) has authorized convalescent plasma therapy and several repurposed drugs (including immune-modulators antivirals and nucleotide analogues) to be used against COVID-19 under certain limited conditions [19,20]. Further, more than nine vaccines have been developed and authorized for human use, varying in mechanism of action and efficacy. Nevertheless, the COVID-19 pandemic is still severe, and most of the currently available antiviral drugs are not designed specifically against SARS-CoV-2. Therefore, the development of new antiviral agents is urgently needed to provide more therapeutic options for managing diseases caused by SARS-CoV-2. However, this strategy became challenging after the emergence of the ability of the virus to mutate into several other forms. Recently, a selection of potential candidates and drugs that could be repurposed for COVID-19 has been reviewed [21,22].

Currently, many medicinal natural products have been identified as displaying a potential antiviral activity against several viruses including hepatitis C virus (HCV), Middle East respiratory syndrome (MERS), influenza viruses, and human immune deficiency virus (HIV). Suwannarach et al., reported the current discoveries on fungi as a potential source for protease inhibitors and highlighted a set of fungal bioactive compounds with immunomodulatory activity as possible prodrugs for treatment of COVID-19 [23]. A recent in silico study by Rangsinth et al. examined a set of 36 natural compounds for their potency as SARS-CoV-2 main protease inhibitors using molecular docking and in silico ADMET analysis [24]. Recent antiviral plant-based research showed that 219 plants from 83 families possess antiviral activities. Among them, 149 plants belonging to 71 families were surveyed and characterized [25]. Further, various plant metabolites have been reported as potential lead antiviral molecules for further medicinal optimization and antiviral drug development [25]. Antimicrobial peptides (AMPs) have been found in both vertebrate and invertebrate animals, existing in the skin, epithelial cells, and blood of vertebrates, as well as in insect hemolymphs and the venomous secretions of bees, wasps, snakes, and scorpions. AMPs inhibit the growth of a wide range of microorganisms including Gram-positive and Gram-negative bacteria, protozoa, yeast, fungi, and viruses (and are not easy to induce drug resistance) [26,27,28,29,30].

In the current review, we discuss the most recent updates about SARS-CoV-2 virus including its classifications and the molecular structures of different virus proteins. We also provide an overview about the different therapeutic approaches for COVID-19 treatment. Further, we introduce and focus on the most recent information about the natural antiviral compounds extracted from algae, fungi, plants, and scorpion venom and their potency against SARS-CoV-2 infection.

## 2. Taxonomy and Structure of SARS-CoV-2 Virus

**Figure 1:**
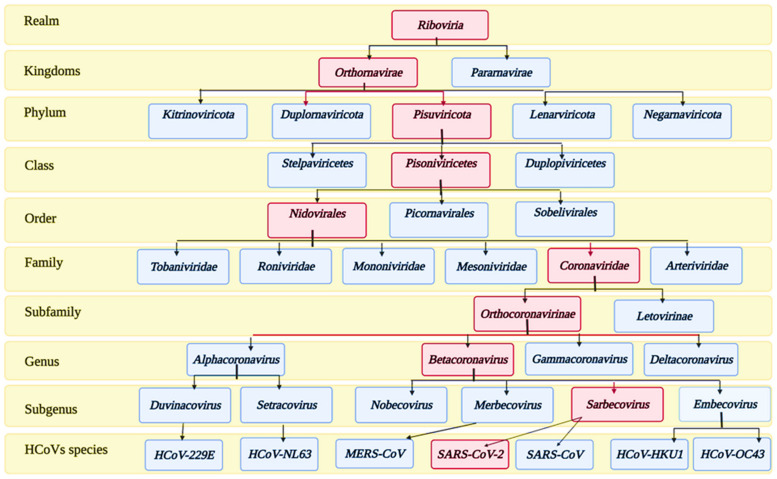
Taxonomy of human coronaviruses.

Coronaviruses (CoVs) belong to the subfamily *Orthocoronavirinae* of the family *Coronoviridae*. The detailed taxonomy of CoVs is illustrated in Figure 1, according to the International Committee of Taxonomy of Viruses (ICTV), where the classification of SARS-CoV-2 appears in the pink squares. Subfamily *Orthocoronavirinae* is classified into four main genera according to the differences in the genomic structure and phylogenetic relationships: *alphacoronavirus*, *betacoronavirus*, *gammacoronavirus,* and *deltacoronavirus*. Mammals are exclusively infected with *alphacoronaviruses* and *betacoronaviruses*, which cause respiratory illness and gastritis in humans and animals, respectively. Birds are infected by gamma and delta coronaviruses, but some can infect mammals as well [31].

Coronaviruses (CoVs) are enveloped and spherical in shape (125 nm in diameter) with an array of projections on the surface that appear as a halo under the electron microscope. The CoV genome is the largest genome among RNA viruses, with a genome size ranging from 26 to 32 kilobases (kb), with a helical-shaped positive sense single-stranded nucleocapsid.

**Figure 2:**
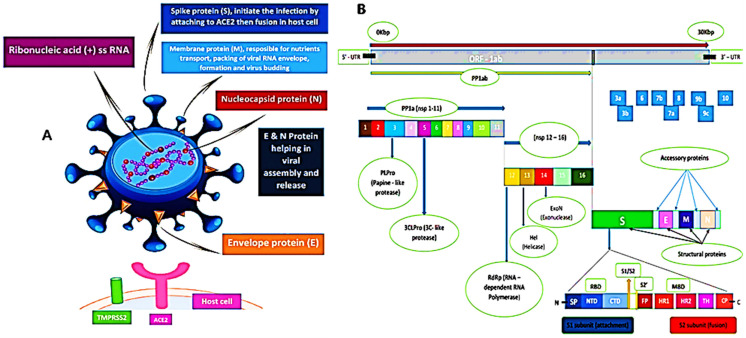
(**A**) 3D graphical presentation of the structure of SARS-CoV-2 and human host cell receptors. (**B**) SARS-CoV-2 genome encodes for 16 nonstructural proteins (nsp), four structural proteins S, M, E, and N, and accessory proteins. A cartoon figure of the SARS-CoV-2 S protein that contains the two subunits: S1 and S2, where S1 composed of: SP (signal peptide); NTD (N-terminal domain), and CTD (C-terminal domain), while S2 composed of FP (fusion peptide), HR1 (heptad repeat 1), HR2 (heptad repeat 2), TM (transmembrane), and CP (cytoplasmic). There are two cleavage sites at S protein denoted as yellow arrows (S1/S2) and (S2′).

The sequence of SARS-CoV-2 showed 88% homology to SARS-like coronaviruses isolated from two bats from Zhoushan, 79% homology to SARS-CoV, and less similarity to MERS-CoV (50%). Interestingly, a computational analysis of SARS-CoV-2 crystal structure showed that SARS-CoV-2 has a binding mode with the host ACE2 receptor similar to that of SARS-CoV and hCoV-NL63 [4,9,15,32,33]. However, the murine monoclonal (mAbs) and polyconal (pAbs) antibodies, raised against SARS-CoV, were not able to inhibit SARS-CoV-2 infection due to the presence of a β1′/β 2′ loop, which is the cause of antigenicity difference [9,34]. As shown in Figure 2, the SARS-CoV-2 S protein binds to the host ACE2 receptor and TMPRSS2, which mediate the viral membrane fusion and initiate the viral life cycle [35,36,37]. The viral RNA replicates uniquely in the cytoplasm of the host cell [38]. The genome of SARS-CoV-2 was sequenced and uploaded to the NCBI genome library (NC 045512.2) (Figure 2) [4]. The genome of SARS-CoV-2 is quite identical to that of SARS-CoV and MERS-CoV, with 14 functional open-reading frames (ORFs) encoding 27 proteins. The two-thirds ORF1ab genome is comprised of 5′-terminal and encoded two huge, overlapped polyproteins, pp1a and pp1ab, that form the viral replicase transcriptase complex [39]. These polyproteins undergo proteolysis by viral proteases (papain-like protease (PLpro) and 3-chymotrypsin-like protease (3CLpro)) to generate 16 nonstructural proteins (nsps), which are highly conserved in CoVs [40,41]. The nsps are essential in viral pathogenesis and involved in many biological processes including, viral entry, replication, protein processing, and the regulation of transcription. On the other hand, the other one-third of ORFs genome encoded the four main structural proteins: spike (S), envelope (E), nucleocapsid (N), and membrane (M) and other accessory proteins [39].

The first Cryo-EM structure conformation of the SARS-CoV-2 S protein was reported by Wrapp et al. [34] and later by Hsieh et al. [42]. The S protein is a 180–200 kDa protein which is responsible for the tissue tropism, attachment of the virus to the host receptors, and viral entry [43]. In addition, S protein mediates the cell-cell fusion and is considered as the highest antigenic target for the host antibody response [44,45]. Investigation of CoVs S protein structure revealed that the S protein is split into S1, S2, and S2′ subunits by the host acid-dependent proteases (mainly, human airway trypsin-like protease (HAT), cathepsins, and TMPRSS2) (Figure 2) [37,46,47,48,49]. The S1 subunit contains a N-terminal domain (NTD) and a C-terminal domain (CTD), and it initiates the infection through binding to the ACE2 receptor on the host cell surface. While the S2 or fusion subunit constitutes different motifs, such as the fusion peptide (FP), the most important functional element for the viral fusion [35,36,37], through the viral fusion process, the S2 protein exists in three different conformations: prefusion native state, prehairpin intermediate state, and postfusion hairpin state [8,34]. There are furin recognition sites between the S1/S2 subunits which are the main factor for the high binding affinity and efficiency of SARS-CoV-2 CTD S protein complex with ACE2 [15,50,51]. Accordingly, furin inhibitors can be considered as potential drug therapies for SARS-CoV-2 [52,53].

The M protein (25–30 kDa) is the most abundant protein which plays an important role in the packaging of the viral RNA and transmembrane-transport of nutrients [49]. The E protein (8–12 kDa) is the tiniest structural protein, and it is crucial for viral assembly and release [11]. The interaction of both M and E proteins defines the viral envelope and helps in the release of virus-like particles (VLPs) [49,54]. The N protein binds to the viral RNA genome and interacts with M and E proteins, which assists the viral RNA packaging, assembly, and budding [55]. Multiple sequence alignment (MSA) revealed that the M, E, and N proteins for BAT-CoV, SARS-CoV, and SARS-CoV-2 are highly conserved and, accordingly, considered as potential drug targets [15,56,57].

The replicase polyprotein plays a crucial role in the virus transcription, translation, and replication, which are also mediated by various functional nsps such as nsp1, nsp2, nsp4, and viral proteinases [58]. Among CoVs, SARS-CoV-2 3CLpro is a highly conserved hydrophilic protein and is considered to be an attractive therapeutic target for SARS-CoV-2 [59,60,61]. In addition, ORF1ab contains a specific RNA-dependent RNA polymerase (RdRp) domain which help in the transcription and replication of the viral RNA and structural proteins (Figure 2) [39]. After assembly, the virions are released via a small vesicle into the host cell surface by exocytosis [62].

## 3. COVID-19 Detection Methods

COVID-19 diagnostic testing is critical for early and accurate detection of the virus, knowing its epidemiology, managing cases, and reducing the risk of spread. To confirm SARS-CoV-2 infection, accurate diagnostic procedures that identify viral nucleic acids, viral antigens, or serological testing are necessary [63]. The presence of illness symptoms is confirmed by chest computed tomography (CT) or magnetic resonance imaging (MRI) [63,64]. For the time being, there are four basic techniques for detecting SARS-CoV-2 infection. The first method needs biosafety level 3 laboratory facilities and involves virus isolation from the patient’s biological materials by using cell cultures. The second is molecular methods such as polymerase chain reaction (PCR), microarray, loop-mediated isothermal amplification (LAMP), clustered regularly interspaced short palindromic repeats (CRISPR), and high-throughput sequencing, which may be used to find viral nucleic acids [65]. The antibody detection by enzyme linked immunosorbent assays (ELISA), immunofluorescence assays (IFA), Western blot (WB) immune-filtration and immunochromatography tests, such as lateral flow immunoassays (LFA), and chemiluminescent immunoassays (CLIA) are the third types of serological testing [66]. Antigen identification with specific monoclonal antibodies to the SARS-CoV-2 antigen is the final step [65]. For SARS-CoV-2 detection, current detection systems employ nasopharyngeal samples; however, oral and blood samples appear to be more suited for future technologies [67].

The WHO has identified the first two molecular diagnostic assays for COVID-19 detection that may be used in an urgent situation to improve illness diagnosis accuracy. The assays for in vitro detection of COVID-19 are real time RT-PCR (qRT-PCR) CoVs and Cobas SARS-CoV-2, qualitative assays for use on the Cobas^®^6800/8800 Systems (Roche Diagnostics, Rotkreuz, Switzerland) [68]. RT-PCR is now the most widely used diagnostic technique for detecting viral RNA through amplification of viral genome. Additional components (probe) are added to situate a foundation that hybridized with the complementary cDNA segment for amplification. The single-step Taqman probe allows real-time quantitative monitoring of the PCR cycle [57]. Nucleic acid detection methods include real-time quantification of the viral genome, which depends on targeting specific regions of the viral genome. Various viral targets include those that are unique to SARS-CoV-2 (such as the viral encoding RdRp gene and the viral N gene) and one that is shared by all members of the Sarbecovirus subgenus (the E gene) [69]. The multiple viral targets were linked to varying levels of specificity and sensitivity, with the E gene being the most sensitive and the RdRp being the most specific [70]. By investigating the released SARS-CoV-2 sequences, specific primers were designed to target the specific genetic regions in the genome of the virus (Table S1). QRT-PCR is a sensitive procedure that only needs a small quantity of viral RNA but takes hours to finish the assay. Unfortunately, such a technique is considered time consuming and requires expensive equipment [70]. Microarray, which relies on the attachment of a viral genome-specific probe, and CRISPR technology, which binds Cas 12/13 enzyme targeted for viral genes for diagnosis of SARS-CoV-2, are two more viral genome-targeting techniques [71].

The Nested RT-PCR procedure was modified to a one-step approach that targeted the ORF1ab and N genes, resulting in a ten-fold improvement in sensitivity over commercial RT-PCR. When compared to standard RT-PCR, the nested RT-PCR demonstrated great accuracy; however, it is likely to provide false negative findings due to crosscontamination that happens during analysis [72]. Among the other nucleic acid procedures are LAMP. It employs the technique of amplifying a specific region of nucleic acid at a particular temperature, providing a quick and accurate detection of SARS-CoV-2. A portable benchtop analyzer proved to be a sensitive, accurate, and powerful instrument for diagnosing SARS-CoV-2, and it could be utilized by workers with no prior PCR experience [73].

The serological technique does not detect the virus; rather, it identifies whether or not someone is infected by detecting an antibody immunological response to previous or current infection [74]. The COVID-19 serological examination has been approved by the European Center for Disease Control and Prevention (ECDCP) for epidemiological and surveillance purposes only [75]. According to research, virus-based IFA and ELISA are extremely sensitive (85–100%) but have poor specificity. The COVID-19 serological test determines the kind and concentration levels of different immunoglobulins in a patient’s serum (IgA, IgM, and IgG) generated as a result of the SARS-CoV-2 infection [66]. Anti-SARS-CoV-2 antibodies levels are linked to illness severity, indicating that individuals with severe illness have a greater viral replication rate and immune activation [76]. False positive findings were caused by antigens that were well conserved across CoV species and crossreaction with autoantibodies in autoimmune disorders, resulting in false positive results [77]. Because both S and N proteins are highly immunogenic, serological tests often identify anti-S or anti-N antibody responses in people with COVID-19 [78]. Additionally, antibody responses to other viral proteins (ORF9b and NSP5) have also been discovered using antibody microarray tests [79]. The data of recent research provide insight into the antibody’s median appearance time in plasma following the beginning of symptoms ranging from 3 to 6 days, and the test accuracy findings remain problematic [80]. IgA could be detected in mucosal secretions within 6–8 days after the infection. IgM takes 3–6 days to appear, while IgG takes 10–18 days, with positive rates of 85.4%, 92.7%, and 77.9% for IgM, IgA, and IgG, respectively, among identified COVID-19 cases [81]. A comparison of the specificities and sensitivities of various serologic diagnostic kits for detection of SARS-CoV-2 antibodies was collected in (Table S2).

Antigen detection methods include the detection of some viral main antigenic proteins, such as the S and N proteins. The S1 subunit is less conserved compared to the S2 unit, but at the same time, it is highly specific to SARS-CoV-2. Thus, it would be a suitable target for serological analysis. In addition, the S1 contains a RBD domain which is highly conserved in the SARS-CoV-2, while the N protein interacts with the RNA and is conserved more than the S protein. The immunochromatographic assay is a popular approach for detecting SARS-CoV-2 antigens [82]. Kits using immunochromatographic techniques showed variable sensitivities and accuracy ranging from 89.2% to 16.7% [83]. Another method, such as biosensors, showed high sensitivity compared to immunochromatographic techniques. They created a cell-based biosensor with a chimeric human spike S1 antibody to detect the SARS-CoV-2 S1 protein, which showed a reliable result for monitoring the SARS-CoV-2 antigens on a large scale [84].

## 4. Molecular Structure and Functional Determinant of SARS-CoV-2

### 4.1. SARS-CoV-2 Proteases

There are two proteases that are encoded in the polyprotein of coronavirus: the main protease (Mpro), also called as 3-C-like protease (3CLpro), and papain-like protease (PLpro) [85]. The two proteases represent crucial drug discovery targets against coronavirus’s family, specially SARS and MERS, and therefore, they were considered to be as potential targets for the most recent SARS-CoV-2 [86,87,88].

#### 4.1.1. Main Protease (Mpro)

The sequence of SARS-CoV-2 Mpro is very similar to that of SARS-CoV with 96.061% identity. On the other hand, the similarity percentage between SARS-CoV-2 Mpro and MERS-CoV is 51.61% [89]. Herein, we have summarized the data for the Mpro protein crystal structure with the highest resolution of resolved structure (Table 1). Generally, the Mpro crystal structure revealed that one polypeptide of the protein forms only one asymmetric unit, which in turn dimerizes upon substrate binding. In this dimer, each polypeptide is called a “protomer”, and each protomer is composed of three domains

(Domain I, II, and III). The domain I is represented by residues 8–101, while the domain II contains residues 102–184. Both domains I and II have an antiparallel β-barrel structure. The domain III, which is composed of residues 201–303, has five α-helices that are arranged into a largely antiparallel globular cluster. Finally, domain III is connected to domain II by a long loop region (residues 185–200). In the cleft between domain I and domain II, the substrate binding region is located and to which the ligand binds. Similar to most of main proteases derived from the corona family, Mpro of SARS-CoV-2 has a catalytic dyad of ‘Cys-His’ [90,91,92,93,94].

**Figure 3:**
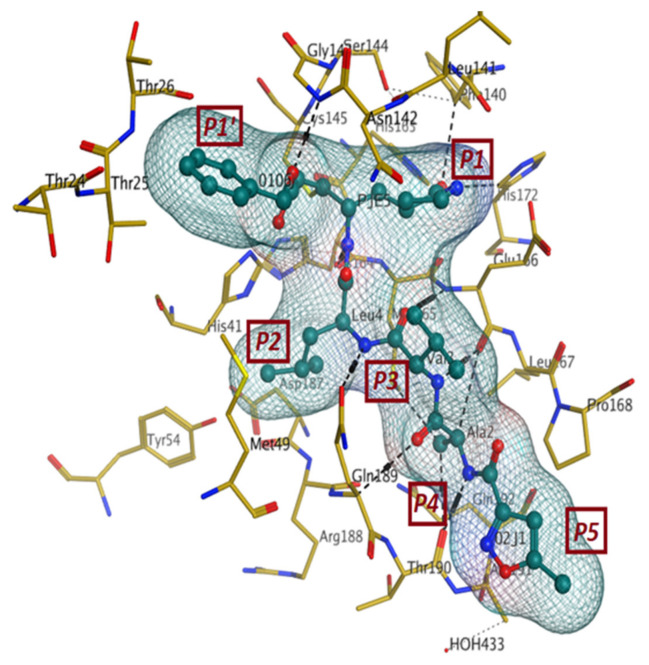
The binding interactions (in black) between N3 inhibitor (in green) and the key residues (in mustard yellow) of the active site in the main protease (Mpro) of SARS-CoV-2 (PDB:6lu7). The inhibitor is divided into 5 parts (P1, P1′, P2, P3, P4, and P5).

The first crystal structure of the SARS-CoV-2 Mpro cocrystallized with N3 inhibitor was recently resolved at a resolution of 2.1 Å (PDB code: 6lu7), which showed the binding of N3 inhibitor to the binding pocket of Mpro protein (Figure 3) [61]. This, therefore, paved the way for designing inhibitors with better affinity. To illustrate the binding interactions between N3-inhibitor and the binding pocket of Mpro protein, the inhibitor has been divided into five parts (given the symbol P). Each part of inhibitor is oriented toward a subunit of the binding site (given the symbol S) to fulfil the interactions between the ligand and the receptor (Figure 3). The atoms of the N3 backbone forms on one side an antiparallel sheet with residues 164–168 and, on the other side, with the part of the loop that connects domain II to domain III (residues 189–191). Additionally, a covalent bond is formed between the CYS145 residue of protomer A and the beta carbon of the vinyl group at N3 scaffold. The lactam ring in P1 has also been involved in the hydrogen bonding with His163 of the S1 in protomer, while the bulky benzyl group extends into the S1′ site and makes Van der Waals contacts with Thr24 and Thr25 of protomer A. The P2 part has a side chain of Leu-residue, which can penetrate the S2 subunit of promotor A. On the other hand, the side chain of the Val-residue in the P3 part is solvent exposed and, thus, can tolerate a wide range of functional groups. In the P4 part, the side chain of the Ala-residue fits in the small hydrophobic pocket of various amino acids. Finally, the P5 part is involved in Van der Waals interactions with Pro168-residue of the protomer A and with the backbone of residues 190 and 191 (Figure 3).

**Figure 4:**
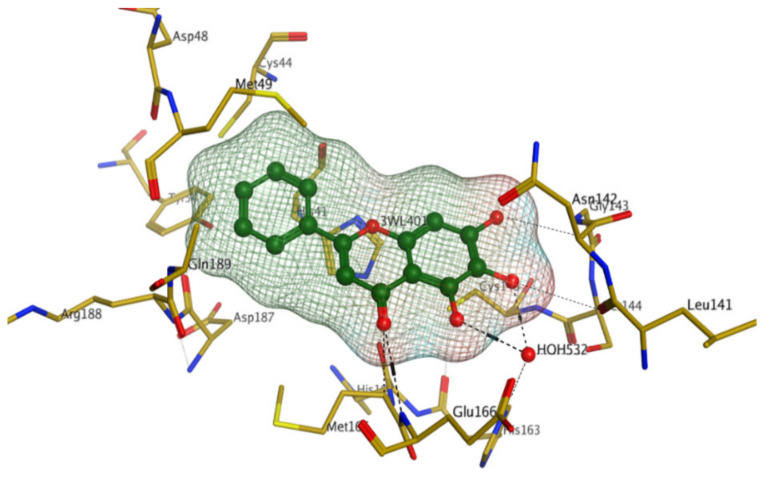
The binding interactions (black-dashed lines) between baicalein (green sticks) and the surrounding key amino acid residues (mustard yellow) of the active site in the main protease (Mpro) of SARS-CoV-2 (PDB: 6M2N).

Later, Xu et al., reported a crystal structure for SARS-CoV-2 3CLpro cocrystallized with baicalin, a bioactive ingredient of Shuanghuanglian, which showed a binding mode distinctly different than the N3 inhibitor (PDB code: 6M2N) [56]. Shuanghuanglian preparation is a Chinese traditional patent medicine (also called proprietary Chinese medicine) used for the treatment of acute respiratory tract infections since 1973, and it is a classical purified herbal preparation extracted from three Chinese herbal medicines; Lonicera japonica Thunb., Scutellaria baicalensis Georgi, and Forsythia suspense (Thunb.) Vahl [56]. In this complex crystal structure, baicalein interacts with the catalytic residues at the core part of the protease substrate-binding region, which is located between domain I and domain II and acts as a shield to hinder the substrate to bind to the catalytic active site. As shown in (Figure 4), baicalein forms a network of hydrogen bonds at the active site through the binding of the phenolic hydroxyl groups to the Ser144/His163 and Leu141/Gly143 residues. Notable, this network of hydrophilic interactions is formed in the presence or absence of the water molecule. Further, the carbonyl group of baicalein forms a hydrogen bond to the Glu166 residue, while the terminal phenyl group is oriented into the S2 subunit and forms a hydrophobic interaction network with His41/Cys44/Met49/Arg188/Gln189 residues. Accordingly, baicalein efficiently binds to the catalytic amino acids residues in the core site and inhibits the protease activity (Table 1) [56].

#### 4.1.2. Papain-like Protease (PLpro)

**Figure 5:**
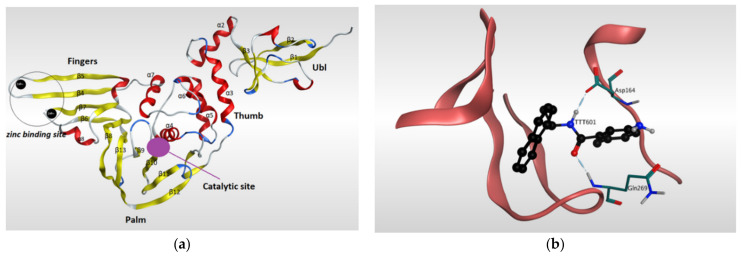
Ribbon representation of Papain-like protease of SARS-CoV-2. (**a**) Ribbon representation of the structure of Papain-like protease of SARS-CoV-2 (PDB:*7CMD*) in its open conformation after removal of the ligand. It illustrates the four subdomains of the enzyme: N-terminal ubiquitin-like domain (Ubl, β1-3), α-helical Thumb domain (α2-7), β-stranded Finger domain (β4-7), and Palm domain (β8-13). Β-sheets are colored in yellow, while α-helices are colored in red. (**b**) Ribbon representation of liganded Papain-like protease of SARS-CoV-2 (PDB: *7CMD*) with GRL0617 and its interaction with the receptor.

Papain-like protease (PLpro) has a significant role in the virus maturation, dysregulation of the immune response, and inflammation mechanism of the host [102]. On the viral scale, PLpro with Mpro hydrolyze the polyprotein into single proteins that are essential for the viral replications. On the host scale, PLpro can act as a protease to cleave peptide bonds and as a deubiquitinating (DUB) enzyme to cleave the isopeptide bonds found in polyubiquitin chains. Recently, the DUB activity of PLpro enzyme showed the ability to attenuate the protective effect of conjugated ubiquitin-like molecules such as the interferon-stimulated gene 15 (ISG15), which in turn helps the virus to maneuver the host’s innate immunity [40,85,103,104,105,106,107]. Because of its dual action on both viral and host sides, PLpro has become an appealing target for SARS-CoV-2. The amino acid sequence of PLpro of SARS-CoV-2 is comparable with that of SARS-CoV with 82% identity and, therefore, the PLpro of both viruses have similarity in most structural features of the orthologs. The resolved 3D-structures of SARS-CoV-2 PLpro are shown in (Table 2). Gao and his colleagues have recently resolved the crystal structure of the unliganded SARS-CoV-2 PLpro by carrying out C-terminal crystallization test of His-tagged C111S PLpro mutant [108]. The results revealed that, compared to PLpro of SARS-CoV, PLpro in SARS-CoV-2 had bigger cell diameters, greater solvent content (around 56%), and distinct crystal packing. The crystal structure of SARS-CoV-2 PLpro has sectioned into four subdomains (Figure 5), the N-terminal ubiquitin-like domain (Ubl, β1-3), the α-helical Thumb domain (α2-7), the β-stranded Finger domain (β4-7), and the Palm domain (β8-13). Additionally, there are four conserved cysteine amino acid residues on the Finger subdomain (Cys189, Cys192, Cys224, and Cys226) which form the zinc binding site (Figure 5) [109]. The PLpro pocket has a catalytic triad that comprises Cys111, His272, and Asp286 residues. It is worth noticing that Cys111 residue, which was mutated to Ser111, is located at 3.6 A° away from the other catalytic histidine H272, and the latter forms a hydrogen bond to the Asp286 catalytic residue at a distance of 3.0 A°. Another significant hydrogen bond is formed between Asp108 and Trp93 residues, which strengthens the oxygen ion hole in the catalytic domain (Figure 5). Upon binding to a ligand, some conformational changes happen to the structure of the protein. For instance, the BL2 loop, which is located between strands β11e-12, changes from a closed conformation to an open conformation by moving 3.2 A° outward in order to adapt the ligand in the binding site [108].

GRL0617 is among the most effective inhibitors of SARS-CoV-2 PLpro [107]. This inhibitor binds to SARS-CoV-2 PLpro in a mechanism that is almost identical to that of SARS-CoV PLpro. GRL0617 fits the gap between the BL2 loop and the loop linking the two loops α3 and α4, mainly occupying the S3 and S4 pockets. By binding to the protein pocket, the inhibitor forms two crucial hydrogen bonds with the receptor: one is formed between the carboxylate side chain of Asp164 residue and N2 nitrogen of the inhibitor, and the other is formed between NH group of Glu269 residue and O7 oxygen of the inhibitor. This H-bonding network has a significant effect of narrowing the cleft between the BL2 loop and the loop connecting α3 and α4, thus preventing any natural ligands from binding with the receptor by clashing with it (Figure 5) [108].

### 4.2. Spike Glycoprotein (S)

The coronavirus spike (S) glycoprotein is the major antigen existing on the surface of the virus. The S-protein is the target of antibodies-neutralization mechanism during infection, and therefore, it is considered as an attractive target for drug design against SARS-CoV-2. The symbol (S) represents a class of viral fusion protein, which is responsible for binding to a target in the host cell, such as angiotensin converting enzyme II in case of SARS-CoV-2. In the S- class, the viral fusion protein starts as a single polypeptide chain template with around 1300 residues, which is then cleaved into two subunits by hosts proteases (S1 and S2). In the prefusion conformation, the two subunits are noncovalently attached [106,111]. SARS-CoV-2 membrane is well known for its club-shaped spikes which are formed by trimers of the S protein [112].

**Figure 6:**
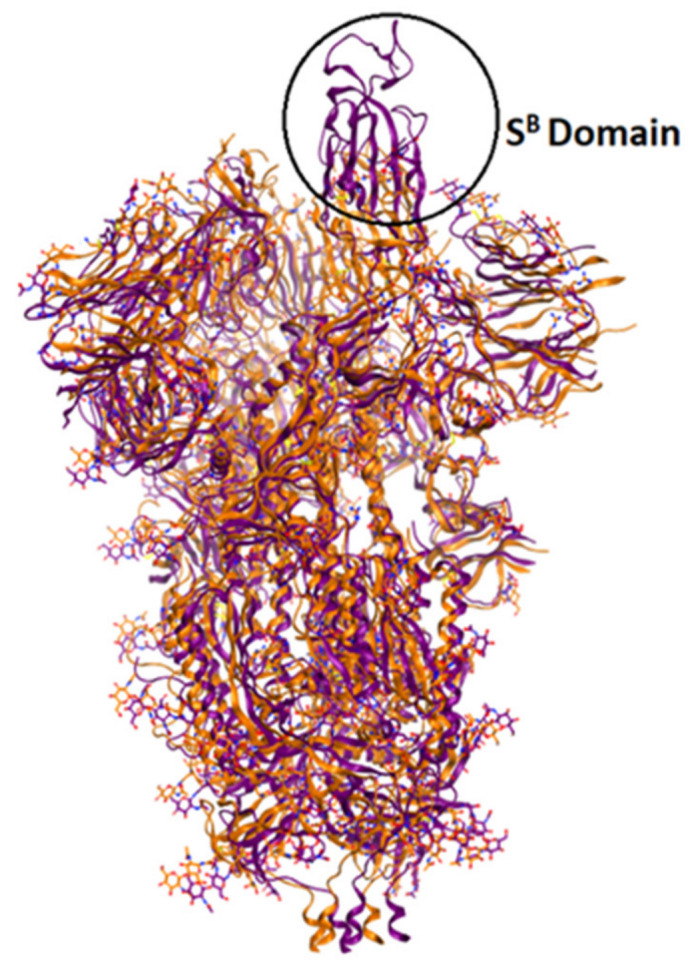
Overlay of ribbon representations of S-glycoprotein of SARS-CoV-2 in open conformation (in purple, PDB: 6vyb) and in closed conformation (in orange, PDB: 6vxx).

The currently available crystal structures of spike glycoprotein are summarized in (Table 3). The crystal structure of SARS-CoV-2 S-glycoprotein revealed that the ectodomain is a 160-A°—long trimer with two subunits (S1 and S2) and a triangular cross-section, which looks very similar to that of SARS-CoV. The S1 subunit is a V—shaped subunit with SB part that changes its conformation to recognize and bind to the host target (Figure 6). The conformation of SB part for this domain has to be in the opening conformation in order to interact with the host target (ACE2) and thus to initiate a series of further conformational changes that lead to cleavage of the S2 subunit, membrane fusion, and finally viral entry [8,112,113,114].

### 4.3. RNA-Dependent RNA Polymerase (RdRp)

**Figure 7:**
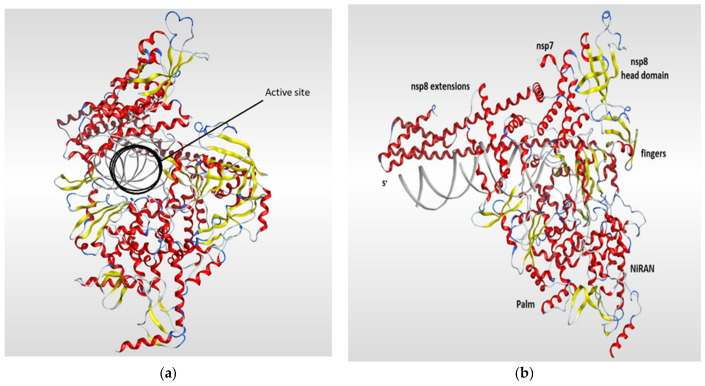
Ribbon representation of RdRp of SARS-CoV-2 (PDB code: 6yyt) which shows the active site of the enzyme (**a**) and illustrates the various domains of the enzyme (**b**). The RdRp structure consists of three subunits (nsp12, nsp8, and nsp7). The RdRp domain is fashioned into three subdomains (palm, fingers, and thumb subdomains). The nsp12 is mainly forms the active site of RdRb and comprised three domains (INRAN domain, C-terminal domain, and interface domain). The nsp8 and nsp7 subunits are binding to the fingers and thumb subdomains.

RNA-dependent RNA polymerase (RdRp), also called RNA replicase, is an enzyme that is encoded in the genome of most of RNA-containing viruses and has a significant role in catalyzing the replication process of the RNA from RNA template [106]. The summary of the known 3D structures of the RdRp is shown in (Table 4). The crystal structure of the RdRp of SARS-CoV-2 cocrystallized with two turns of RNA duplex was resolved (PDB code: 6YYT). The SARS-CoV-2 RdRp structure is similar to that of the SARS-CoV RdRp but with an extra protrusion that fits the RNA duplex. As shown in (Figure 7), the RdRp structure consists of three viral protein subunits, nonstructural protein 12 (nsp12), nsp8, and nsp7 together with RNA template–product duplex. While nsp7 and nsp8 are acting as accessory subunits, nsp12 comprised three domains: mainly an interface domain, an N-terminal nidovirus RdRp-associated nucleotidyltransferase (NiRAN) domain, and a C-terminal domain. The active site is located in the palm subdomain and is composed of five conserved nsp12 elements that are known as motifs A–E. Motif C, which is formed by the essential residues Asp760 and Asp761, binds to the 3′ end of the RNA. Two additional motifs, F and G, were retained at the fingers subdomain and postured the RNA template. One main interaction between the RNA and the RdRp is that one formed between the first turn of RNA and the nsp12 subunit of the enzyme between its fingers and thumb subdomains. The protruding RNA duplex is sandwiched by long α-helical extensions that are produced by positively charged residues that cover up to 28 base pairs running away from the active site and interact with the backbone of RNA. Those extensions are formed by highly conserved N-terminal regions in two nsp8 subunits which in turn differ according to their RNA interactions (Figure 7) [117].

### 4.4. SARS-CoV-2 Nucleocapsid N Protein

**Figure 8:**
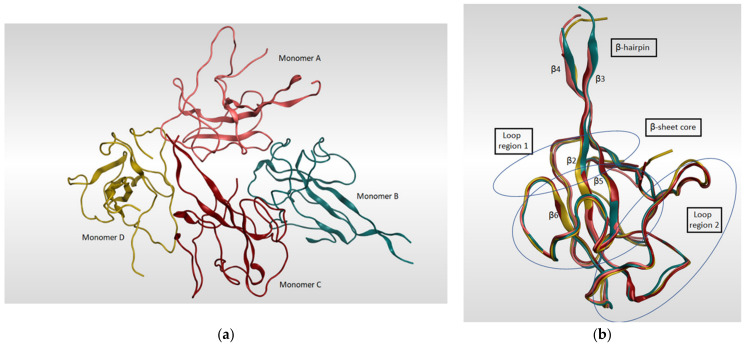
(**a**) Ribbon representation of SARS-CoV-2 nucleocapsid protein N-terminal RNA binding domain (PDB: *6M3M*) which shows four monomers in an asymmetric unit, each colored in different color. (**b**) illustrates the four monomers superimposed on each other and shows the sandwich effect of two loop regions on the β-sheet core.

The SARS-CoV-2 nucleocapsid N protein is considered to be the only structural protein that is related to the replicase–transcriptase complex (RTC), as it binds to gRNA and plays an important role in the incorporation of the virus genetic material into coronavirus’s particles. Furthermore, it has a crucial role in designing the architecture of the virus particles through an interaction network with gRNA, M protein, and other N molecules [118]. The genomic sequence of the N-protein encoding region in SARS-CoV-2 N was found to be very similar to that in SARS-CoV with an identity percentage of 89.74% [119,120]. Since there are two ways of N-protein packing for crystallization [121], monoclinic and cubic, there might be an implication of the potential contacts in SARS-CoV-2 RNA N-protein formation process. A summary of the known 3D structures of SARS-CoV-2 nucleocapsid N protein is detailed in Table 5. In a study reported by Kang et al., the crystal structure of N-terminal RNA-binding domain (NTD) revealed that it packs into an orthorhombic crystal form, where the interfacial interactions are produced by residues of β-hairpin fingers and palm regions [122]. Moreover, one asymmetric unit of SARS-CoV-2 N-NTD consists of four monomers which share similar right-handed and sandwiched pattern of (loops)-(β-sheet core)-(loops) (Figure 8). The core pocket is composed of five antiparallel β-strands with a single short helix, which is located before strand β2, and a protruding β-hairpin between strands β2 and β5. In addition, the protein is enriched in aromatic and basic amino acids, which are folding and shaping a right-handed shape. This is in turn similar to the structure of a protruding basic finger, a basic palm, and an acidic wrist [122].

## 5. Therapeutic Approaches for COVID-19

### 5.1. Antiviral Strategies against SARS-CoV-2

Direct-acting antivirals (DAA) and indirect-acting antivirals (IAA) are the two types of antivirals available. Viral polymerase is one example of a specific viral ingredient that DAAs target without interfering with the normal functioning of the host cellular systems. The progress of DAAs can facilitate the treatment of patients with COVID-19. IAAs, on the other hand, target host proviral factors and indirectly decrease viral replication by interfering with their activity or interaction. IAAs offer a distinct advantage over DAAs in that they are not susceptible to viral mutations, which are common in RNA viruses. However, IAA can alter the host’s biological processes and are therefore not regarded as safe. As a result of their greater safety features, DAAs that target viral entry, proteases, and replication could be useful as antivirals. Because there is no licensed antiviral medication for SARS-CoV-2, drug repurposing of previously used antiviral medicines is one of the most widely used techniques. In addition, the de novo development of drugs costs over $1 billion USD and 10–17 years [125]. Several authorized antivirals have been repurposed to treat COVID-19. Various existing broad-spectrum antiviral medicines (BSAAs) have been extensively tested in clinical trials; for instance, phase II umifenovir is an indole-based antiviral medication that is used in Russia and China to treat influenza, and umifenovir’s antiviral activity is suggested to be linked to interactions between its aromatic residues and viral glycoproteins, which are involved in viral adhesion through the cell membrane. Lopinavir/ritonavir is a drug combination targeting viral protease, both approved for the indications of and HIV and influenza [126]. They are considered in phase IV clinical trial for pneumonia associated with COVID-19 (ClinicalTrials.gov ID: NCT04255017) [127]. At the phase III level, remdesivir (RDV), a nucleotide analogue inhibitor of RdRps and a broad-spectrum antiviral medication discovered in 2014 for the treatment of Ebola virus (EBOV), United States FDA has approved the use of remdesivir for COVID-19 infection. Remdesivir inhibits replication and has demonstrated efficacy in the treatment of COVID-19 at late stages. It is combined into coronavirus single-stranded RNA via polymerase enzyme, inhibits the addition of new RNA subunits, and restricts genome replication [128]. Remdesivir is under investigation for mild and moderate SARS-CoV-2 (ClinicalTrials.gov ID: NCT04252664) [129]. Recently, through a compassionate-use indication, remdesivir has supportive evidence for yielding some clinical improvement in COVID-19 patients [130]. In addition, a temporary analysis of the adaptive COVID-19 treatment trial (NCT04280705) supports improvement in the primary endpoint for patients receiving remdesivir, compared to control, with a 31% faster time to recovery [131]. Other phase III antivirals being estimated in combination therapy for viral pneumonia interestingly include the antimalarial hydroxychloroquine, based on promising in vitro data (ClinicalTrials.gov ID: NCT04261517) [132]. Chloroquine, in addition to its immunomodulating properties, has been revealed to have an antiviral effect at entry and postentry stages of the SARS-CoV-2 infection. It can boost the antiviral activity of remdesivir and potentially serve as a synergizer of BSAAs [125]. As a purine nucleotide, favipiravir is capable of inhibiting RdRp and so preventing viral generation. It is authorized in Japan and China for the treatment of new influenza viruses and has antiviral action against a wide variety of RNA viruses [133]. FDA-approved alcoholism medicine disulfiram has been generally acclaimed for its ability to block the MERS and SARS PLpro and could now play a role in the fight against SARS-CoV-2. In SARS-CoV-2, disulfiram prevents the release of viral genome by inhibiting the papain-like protease [134]. Disulfiram has the ability to reduce the hyperinflammatory response caused by COVID-19. The medicine suppresses the production of gasdermin D pore, which reduces pyroptosis and netosis and may be used to tackle the root cause of hyperinflammation, decreasing the cytokine storm and thereby lowering the risk of severe infection (ClinicalTrials.gov ID: NCT04594343) [132]. However, the usage of DAA may increase the chance of drug-resistant mutations, and a combination of repurposed therapies can shorten treatment time, lower treatment costs, lower the risk of drug resistance, and improve therapeutic efficiency, making it easier to enter clinical trials [135]. Furthermore, there is also the possibility to design medications with lower off-target toxicity by using crystal structures of viral and host cellular proteins associated with SARS-CoV-2, as S protein, Mpro, RdRp, and hACE2 [136].

### 5.2. Immuno-Modulators

A high concentration of proinflammatory cytokines was found in the initial report of pathological characteristics of a patient who died from severe SARS-CoV-2 infection. Genuinely, in a large group of critically sick patients infected with COVID-19, cytokine storms triggered by the overproduction of proinflammatory cytokines have been observed [137]. Patients who have experienced cytokine storms experience multiple-organ failure and die quickly. Thus, the early detection, treatment, and control of cytokine storms are significant for patients [138]. Interleukin-6 (IL-6) is a cytokine involved in inflammatory and immunological responses [139]. Tocilizumab (TCZ), a humanized monoclonal antibody, is anti-interleukin-6 receptor (IL-6R) and is suggested in critically ill patients with increased IL-6 levels. TCZ inhibits cytokine storms and may help to stabilize patients’ conditions [140]. Corticosteroids are also utilized in the treatment of COVID-19, and they inhibit proinflammatory storms, particularly in the lungs [141]. Dexamethasone is a highly active antiedema and antifibrotic agent. The administration of dexamethasone via intravenous injection or inhalation may help to improve anti-COVID-19 treatment effectiveness by targeting the potent corticosteroid drug to hyperactivated immune cells, by potentiating its antiedema action and by exploiting its antifibrotic effects [142]. Another potential immunomodulator in COVID-19 treatment is hydroxychloroquine (HCQ), and the primary use of HCQ, beyond its well-known role as an antimalarial drug, is as an immunomodulator for autoimmune syndromes such as systemic lupus erythematosus (SLE). Treatment with HCQ modifies the n-terminal glycosylation of ACE2, which decreases the sensitivity of ACE2-S1 (Spike) interaction [143]. HCQ can prevent viral infection by altering endosomal acidification to restrict viral adherence; in addition, HCQ suppresses lysosomal antigen processing by antigen-presenting cells and lowers T-cell adhesion, and the consequent generation of pro-inflammatory cytokines involving TNF-α and IL-6, the impact of HCQ on cytokine production, and suppression of antigen presentation may have immunologic ramifications that obstruct antiviral immune reactions for COVID-19 patients [144]. Although ACE2 receptors have been known as the key receptors for the entrance of SARS-CoV-2, it was discovered that SARS-CoV-2 also targets cells without ACE2 receptors, such as lymphocytes. Inhibiting clathrin-mediated endocytosis may be effective in preventing SARS-CoV-2 from entering the cell. A putative target for SARS-CoV-2 infection is the Janus-associated kinase (JAK), which is one of the primary regulators of endocytosis [145]. The JAK inhibitors ruxolitinib, fedratinib, upadacitinib, tofacitinib, and filgotinib are used to treat myelofibrosis and other inflammatory diseases. Myelofibrosis is a blood cancer characterized by chronic leukemia. Lymphocytopenia is a common symptom in COVID-19 patients as well and is considered as one of the main markers of the disease. Therefore, it is suggested to repurpose JAK inhibitors in COVID-19 patients, since the disease resembles the myelofibrosis symptoms. Furthermore, JAK inhibitors have powerful anti-inflammatory characteristics and can help COVID-19 patients avoid a cytokine storm, and JAK inhibitors are also regarded to be reasonably safe therapies for SARS-CoV-2 because they block inflammatory mediators such as INF-α, which are important in immune responses [141]. Another JAK inhibitor, baricitinib, has been recommended as the best choice among other JAK inhibitors due to its tolerable side effect profile, the potential of once-daily dose, better efficacy, and favorable pharmacokinetics. Baricitinib inhibits cyclin-G-associated kinase, which is endocytosis regulator, through which it can defeat the viral infection [146]. Tacrolimus, a calcineurin inhibitor, which is mainly used in organ transplantation, was revealed to be effective against MERS-CoV in a renal transplant patient. In a cell line investigation, tacrolimus was also reported to be effective against SARS-CoV. However, more research is needed to determine its effectiveness against SARS-CoV-2 [147]. Sirolimus is an immunosuppressive medicine in which mTOR inhibitors were discovered to limit memory B-cell activation and block the antibody-dependent enhancement mechanism. The inhibitors of mTOR were recorded to inhibit the replication of MERS-CoV in the in vitro studies. Sirolimus was revealed to constrain viral replication in patients with acute respiratory failure [148].

### 5.3. Antibody and Convalescent Plasma Therapy

The transfusion of neutralizing antibodies obtained from a hyperimmune patient is the most common and accessible empirical strategy used to treat a wide range of viruses and other infectious diseases. This procedure, known as convalescent plasma treatment (CPT), is also thought to be an effective COVID-19 treatment [149]. It has been demonstrated to be effective in reducing the period of stay in the hospital and the mortality rate of hospitalized patients with severe acute respiratory syndrome [150]. This method was proposed for the first time during the Spanish influenza outbreak [149]. Following that, plasma transfusion was recommended as a safe and effective strategy to prevent or treat Ebola in 2014, as well as several other serious viral diseases such as MERS, SARS-CoV, and avian influenza A [151]. Evidently, neutralizing antibodies in convalescent plasma (CP) could diminish viral load by binding to the viruses’ surface antigens and blocking virus entry into host cells [151]. CPT efficacy may differ depending on the type of microorganism, its pathogenicity, and treatment strategies such as timing, dose, and volume of injection. Early CP transfusion is expected to be more beneficial and enhance the survival rate of critical COVD-19 patients at the early disease stage, as per previous evidence for plasma therapy of other coronaviruses, such as SARS-CoV and MERS.

It could be explained by the fact that in several viral infections, the number of cases increases during the first week of the disease [152]. The investigation focuses on the virus’s mode of action. According to Ling Lin et al., the virus first attacks organs in patients who express ACE2 receptors, followed by a second attack 7–14 days later [153]. In disease progression, the virus may produce a decrease in B lymphocytes (and IL-6 reduction), which could affect antibody production. Lymphocytes may continue to decline as the disease progresses, but inflammatory cytokines increase as well. Therefore, treatments should focus on (1) improving patients’ immunological function and (2) suppressing the cytokine production [154]. The instant use of convalescent plasma affords the immediate access of a promising treatment, while vaccination and treatments are studied and scaled up. Using convalescent plasma from donors who have recently recovered from COVID-19 may be most promising when used as prophylactic or when injected shortly after symptoms begin (within 14 days). The protection may last from weeks to months [155].

### 5.4. COVID-19 Vaccines

Safe and effective vaccines against SARS-CoV-2 are key to overcoming the global pandemic. New vaccine development typically takes >15 years, but SARS-CoV-2 vaccines are being tested on an unparalleled fast track [156]. Many have estimated that SARS-CoV-2 vaccines could be developed in as short as 15–18 months [156]. There are numerous vaccination strategies tested in animals that can be categorized into five different platforms: live attenuated viruses, nucleic acid vaccines (mRNA and DNA), viral vectored vaccines, and protein subunit vaccines, and each platform has its strengths and limitations [157]. Most of the anti-COVID-19 vaccine safety studies exclude elderly adults due to increased morbidities and weakness such as AstraZeneca, Moderna, and Pfizer have rare data concerning vaccines safety in aged and sensitive peoples (Table 6) [141].

#### 5.4.1. BNT162B1 (Pfizer-BioNTech)

mRNA and DNA vaccines represent a promising alternative to conventional vaccine approaches because of their high stability, high potency, capacity for rapid development, and potential for low-cost manufacture and safe administration [158]. A lipid nanoparticle-formulated, nucleoside-modified, mRNA vaccine that encodes trimerized SARS-CoV-2 spike glycoprotein RBD, BNT162B1, attacked the RBD of the S protein in phase I/II trials. The majority of vaccine recipients had mild to moderate systemic and local symptoms, and transient RBD-binding IgG concentrations and SARS-CoV-2 neutralizing titers in sera increased with administration and after a second dose. The geometric mean neutralizing titers of a sample of COVID-19 convalescent human serum were 1.8–2.8-fold higher. These data suggest that this mRNA vaccine candidate should be further investigated [159]. In the phase III clinical trial, a total of 43,548 participants were randomized, of whom 43,448 received injections: 21,720 with BNT162b2 and 21,728 with placebo. A two-dose treatment of BNT162b2 conferred 95% protection against COVID-19 in people 16 years of age or older. Safety over a median of 2 months was similar to that of other viral vaccines. BNT162B1 funded by BioNTech and Pfizer; ClinicalTrials.gov ID: NCT04368728 [160]. BioNTech (Pfizer) is the first safe and efficacious COVID-19 vaccine to be approved for emergency use, while WHO does not recommend it for children under the age of 16. Further, there is a scarcity of safety information for pregnant or breastfeeding women [141].

#### 5.4.2. CoronaVac (SinoVac)

Sinovac vaccine denatured with aluminum hydroxide has proceeded to a phase III clinical trial. The vaccine is well tolerated and immune stimulating in healthy adults, according to Zhang et al. To determine the optimal dose, immunogenicity, and safety of the CoronaVac, researchers conducted a randomized, double-blind, placebo-controlled trial. A total of 600 healthy adults aged 18–59 years were assigned to receive two injections of the trial vaccine at a dose of 3 μg/0.5 mL or 6 μg/0.5 mL, or placebo on the day 14 schedule or day 28 schedule. CoronaVac conferred 50% protection against COVID-19 patients. (ClinicalTrials.gov ID: NCT04352608) [161].

#### 5.4.3. mRNA1273 (Moderna)

The nucleoside-modified messenger RNA (modRNA) encoding the viral spike (S) glycoprotein of SARS-CoV-2 is formulated in lipid particles, which enable delivery of RNA into host cells to allow the expression of the SARS-CoV-2 S antigen, and stimulates an immune response to the S antigen, which protects against COVID-19. Jackson and his colleagues conducted a first-in-human phase I clinical trial in healthy adults to estimate the safety and immunogenicity of mRNA-1273. Contributors were 18–55 year old adults who received two injections of trial mRNA-1273 vaccine 28 days at a dose of 25, 100, or 250 μg. After the first vaccination, antibody responses were higher with 250 μg dose, and after the second vaccination, the antibody titers were amplified. Serum-neutralizing activity was evaluated by two procedures in all contributors. They recorded that the two-dose vaccine sequence could trigger neutralization and Th1-biased CD4 + T-cell responses. These findings support the further advancement of this vaccine. (mRNA-1273 ClinicalTrials.gov ID: NCT04283461) [162]. The phase II clinical trial conducted by Chu et al. recorded that the vaccination with mRNA-1273 resulted in significant immune responses to SARS-CoV-2 in participants 18 years and older, with an acceptable safety profile, approving the safety and immunogenicity of 50 and 100 µg mRNA-1273 given as a two-dose regimen. mRNA1273 total efficacy is 94.1% (ClinicalTrials.gov ID: NCT04405076). In clinical trials, the majority of side effects that occur within 7 days post vaccination are mild to moderate. Few people had reactions that affected their ability to perform daily activities.

#### 5.4.4. Ad26COVS1 (Jansseen Vaccine)

The Ad26.COV2.S (Janssen Vaccines) is a recombinant transgenic vaccine, replication-incompetent human adenovirus type 26 vector encoding full-length SARS-CoV-2 spike protein in a prefusion-stabilized conformation. Sadoff et al. conducted a phase III clinical trial, and Ad26.COV2.S was given to 19,630 SARS-CoV-2–negative participants, while a placebo was given to 19,691. Ad26.COV2.S provided protection against moderate to severe–critical infections. COVID-19 has an onset at least 14 days after the dose and a duration of at least 28 days. The vaccine exhibited 73.1 in severe and 81.7% in critical COVID-19, respectively. Ad26.COV2.S had a higher rate of reactogenicity than placebo but was mild to moderate. They concluded that a single dose of Ad26.COV2.S protected against symptomatic and asymptomatic SARS-CoV-2 infection and was effective against severe–critical disease, including hospitalization and death [163]. Safety appeared to be similar to that in other phase III trials of COVID-19 vaccines, funded by Janssen Research and Development and others, ENSEMBLE (ClinicalTrials.gov ID: NCT04505722) [164]. WHO recommends the Janssen (J and J) vaccine for people aged 18 and older. Pregnant and lactating women can be vaccinated with J&J on the basis of risk–benefit ratio. It is not recommended for hypersensitive individuals or those with a history of allergies to a component of the vaccine.

#### 5.4.5. ChAdOx1 nCoV-19 (AstraZeneca)

The AstraZeneca/Oxford product is a viral vectored vaccine called ChAdOx1 nCoV-19/AZD1222 vaccine consists of a replication-deficient chimpanzee adenoviral vector ChAdOx1, comprising the SARS-CoV-2 structural surface glycoprotein antigen (spike protein; nCoV-19) gene. Voysey et al. conducted a trial, and 23,848 contributors were enrolled, and 11,636 contributors were included in the efficacy analysis. In contributors who received two standard doses, the vaccine efficacy was 62.1% and in contributors who received a low dose followed by a standard dose, the efficacy was 90.0%. Overall, the vaccine across both groups was 70.4%. From 21 days after the first dose, ChAdOx1 nCoV-19 has an acceptable safety profile and has been found to be efficacious against symptomatic COVID-19 in this interim analysis of ongoing clinical trials, (ClinicalTrials.gov ID: NCT04324606, NCT04400838, and NCT04444674). The vaccine is not recommended for people younger than 18 years of age pending the results of further studies [165].

#### 5.4.6. SputnikV (Gam-COVID-Vac)

The vaccine employs a heterologous recombinant adenovirus technology using adenovirus 26 (Ad26) and adenovirus 5 (Ad5) as vectors for the expression of the SARS-CoV-2 spike protein [166]. In the Lancet, Denis Logunov and colleagues publish input data from a phase III study of the Sputnik V COVID-19 vaccine. A total of 21,977 people were randomly assigned to one of two groups: the vaccine group (*n* = 16,501) or the placebo group (*n* = 5476). The primary outcome analysis included 19,866 people who received two doses of vaccination or a placebo. From 21 days after the first dose of vaccine, 16 (0.1%) of 14,964 participants in the SputnikV vaccine group and 62 (1.3%) of 4902 in the placebo group were confirmed to have COVID-19; total vaccine efficacy was 91.6%. With the exception of a rash and an immunological reaction, there are no severe side effects. In the vaccination community, three severe adverse effects were identified in the vaccine group among participants above the age of 60 years experiencing renal colic, deep vein thrombosis, and abscesses. This trial is registered with (ClinicalTrials.gov ID: NCT04530396) [167].

#### 5.4.7. BBIBP-CorV (Sinopharm)

Inactivated viruses can produce local antigenic epitopes. These viral-neutralizing epitopes bind to T- and B-cell antibodies and are present in a stable mode. In these vaccines, aluminum hydroxide is utilized as an adjuvant to strengthen the host’s immune system for combination vaccines [168]. Beijing Bio-Institute of Biological Products produces BBIBP-CorV (BBIBP). SARS-CoV-2 is chemically inert in the BBIBP-CorV vaccine; therefore, it cannot replicate, but the entire protein is still integral. Xia and his colleagues conducted a phase I/II clinical trial of this vaccination in comparison to a placebo control in Shangqiu City, China. In total, 1120 people between the ages of 18 and 59 and 608 people over the age of 60 were tested. The initial findings of the phase I/II experiment revealed that the inactivated vaccination against SARS-CoV-2 was safe and immunogenic in adults, including those aged 60 and older. All tested dosages demonstrated 79% efficacy against COVID-19. This study is registered with www.chictr.org.cn, accessed on 29 April 2020, ChiCTR2000032459 [169]. The Sinopharm Vac. (BBIBP-CorV) is still not approved by the world’s drug regulatory agencies, including the European Medicine Agency (EMA), the FDA, and the Medicines and Healthcare products Regulatory Agency (MHRA). On 7 May 2021, the WHO approved its usage for emergency purposes in people over the age of 18. Minor side effects of Sinopharma Vac. in people aged 19–59 include fever, allergies, pain, headache, and swelling at the injection site, while major side effects include nausea, facial nerve symptoms, clot formation, and acute disseminated encephalomyelitis [170].

#### 5.4.8. NVX-CoV2373 (Novavax)

This recombinant protein vaccine uses various versions of the S-protein as its vaccine antigen component. The NVX-CoV2372 trimeric nanoparticle produced by Novavax is made from the full-length S-protein. In its phase I/II study, Novavax’s NVX-CoV2373 vaccine, formulated with Matrix-M, produced a Th1-biased immune response. Novavax’s proprietary Matrix-M adjuvant consists of two individually nanosized particles. Matrix-M has been proven to augment both Th1 and Th2 type responses, inducing high levels of neutralizing antibodies and enhancing immune cell trafficking [171]. Researchers estimated that Novavax has 96% efficacy in COVID-19 patients under clinical trial phase III. Headache and muscle ache were the most commonly reported side effects among vaccination recipients (ClinicalTrials.gov ID: NCT04611802). Novavax has developed agreements with several manufacturers comprising Emergent, Fujifilm, AGC Biologics, and the Serum Institute of India to produce 2 billion doses annually [172].

#### 5.4.9. BBV152 (Covaxin)

It is also known as Covaxin and is manufactured by Bharat Biotech, India. A whole-virion-inactivated SARS-CoV-2 vaccine was formulated with a Toll-like receptor 7/8 agonist molecule adsorbed to alum (Algel-IMDG) or alum (Algel). Ella and his colleagues tested BBV152′s safety and immunogenicity in 11 hospitals across India in a random and controlled phase I experiment. A total of 827 people were investigated; among the registered participants, 100 were each randomly assigned to the three vaccine groups, and 75 were randomly assigned to the control group (Algel only). The most common systemic side effects were injection site pain, headache, fatigue, fever, and nausea after two doses. All adverse effects were mild or moderate and were more frequent after the first dose. The trial is registered at (ClinicalTrials.gov ID: NCT04471519). BBV152 induced binding and neutralizing antibody responses and with the inclusion of the Algel-IMDG adjuvant. BBV152 exhibited an 81% efficacy against the COVID-19 original strain [173].

#### 5.4.10. Ad5-nCoV (CanSino)

Ad5-nCoV was developed by the Beijing Institute of Biotechnology, Beijing, China, and CanSino Biologics, Tianjin, China. It is single-shot vaccine with similar efficacy to other vector vaccines such as J&J, Gamaleya, and AD26. It is suggested for people 18 years of age and above [174]. Wu and his colleagues reported the safety, tolerability, and immunogenicity of an aerosolized Ad5-nCoV in adult, and they stated that the aerosolized Ad5-nCoV is well tolerated, and two doses of aerosolized Ad5-nCoV produce neutralizing antibody responses, similar to one dose of intramuscular injection. An aerosolized booster vaccination at 28 days after the first intramuscular injection stimulated strong IgG and neutralizing antibody responses. The humoral and cellular immune responses induced by aerosolized Ad5-nCoV, and its dose-sparing potential shows that aerosol vaccination is a promising method for the delivery of the COVID-19 vaccine. Ad5-nCoV exhibits 65.7% efficacy in COVID-19 patients. This trial is registered with (ClinicalTrials.gov ID: NCT04552366) [175].

## 6. Natural Products and SARS-CoV-2

### 6.1. Antiviral Natural Products from Fungi

Fungi are a rich and potent reservoir of different biomolecules, which can serve as a sustainable source of novel therapeutic ingredients [176]. Bioactive myco-compounds showing antiviral activities are now being investigated, and the amount of research is steadily growing [177]. Fungal biomolecules can be categorized into two main groups; low-molecular-weight compounds generated by filamentous fungi, including endophytic fungi, and high-molecular-weight compounds obtained from the mycelia and fruiting bodies of medicinal mushrooms [178]. Fungal indole alkaloids, nonribosomal peptides, polyketides, hybrids of nonribosomal peptides and polyketides, as well as terpenoids, have been recognized as low-molecular-weight molecules with considerable antiviral properties [179]. Other antiviral high-molecular-weight molecules have been isolated from mycelia and fruiting bodies of medicinal mushrooms, viz: lignin derivatives, polysaccharides (viz, lentinan, chitin, and mannan), polysaccharide-protein/amino acid complexes, and proteins [23].

In general, the suppression of viral replication is a major challenge and the main goal for discovering new drugs where protease enzymes are required by a variety of viruses for replication, transcription, and maturation [180]. As a result, numerous investigations have focused on the identification of a protease inhibitory target that is required for viral transcription and replication [178]. Two proteases (3CLpro and PLpro) have been considered in CoVs as promising therapeutic drug targets for viral inhibition [181]. Fonsecin is a naphthopyrone pigment that was discovered in an *Aspergillus fonsecaeus* mutant. The crude pigment may be readily removed from dried fungus mycelium using ethyl acetate. Based on in silico molecular docking and molecular dynamics studies, Fonsecin has a high binding affinity for SARS-CoV-2-PLpro by interacting with the Tyr268 amino acid residue of the enzyme cavity [182]. The genome of *Penicillium thymicola* contains a polyketide synthase and a nonribosomal peptide synthetase hybrid gene cluster, which upon expression leads to the synthesis of Pyranonigrin A. Pyranonigrin A. is a secondary fungus metabolite with strong inhibitory capability against the SARS-CoV-2 Mpro. An in silico modeling study showed that Pyranonigrin A is capable of forming seven hydrogen bonds on par with the N3 inhibitor and is also expected to create a covalent bond with Mpro [183]. A computational study of bergenin, quercitrin, and dihydroartemisinin purified from *Dictyophora indusiata*, *Geastrum triplex*, and *Cyathus stercoreus*, respectively, was assayed based on their medicinal uses [184]. Bergenin is a C-glucoside of 4-O-methyl gallic acid that has been utilized as a traditional remedy in several Asian countries for many years [185]. Bergenin has antiparasitic, antiviral, anti-HIV [186], immunomodulatory, and anti-HCV properties [187]. The glycoside quercitrin is made up of the flavonoid quercetin and the deoxy sugar rhamnose. Quercitrin inhibited HIV-1 reverse transcriptase [188] and had an antiviral effect against infection with the HCV [189] and dengue virus [190]. Dihydroartemisinin is a water-soluble artemisinin derivative that is a safe and effective antimalarial medication [191]. In an in-silico investigation, dihydroartemisinin was found to be a potent inhibitor of SARS-CoV-2 M^pro^, indicating that it might be a viable molecule against SARS-CoV-2. However, more research is needed to demonstrate its therapeutical application [184]. Drugs that inhibit viral proteases, such as HIV-1 protease inhibitors and HCV NS3/4A protease inhibitors, have been considered effective and promising prodrugs against CoV infection [23].

Pyrrocidines A, a polyketide-amino acid-derived antibiotic, is produced from the endophytic fungi *Acremonium zeae* [192]. Dankasterone B is produced from the endophytic fungus *Gymnascella dankaliensis*, derived from *Halichondria sponge* [193]. A computational study using molecular docking and molecular dynamic simulation found that pyrrocidine A and dankasterone B, secondary metabolites of fungi, are potent inhibitors of viral RdRp and can be exploited in further research to develop efficient anti-coronavirus drugs [194].

One of the primary complications of COVID-19 disease is the hyperinflammatory response and cytokine storm correlated with higher immune activation [195]. Accordingly, immunosuppressants have been considered in treating COVID-19 patients to avoid hyperactivation [195]. Cyclosporine, isolated from the fungus *Beaueria nivea*, is an inhibitor of cyclophilin that also targets calcineurin. It creates a cyclosporine-cyclophilin complex with the cyclophilin receptor in cells, which blocks the calcium-dependent interleukin (IL-2) synthesis pathway, inhibits IL-2 gene transcription, and decreases inflammatory reactions [196]. Cyclosporine A, an authorized immunosuppressant, is also used to treat hepatitis C [197] and MERS-CoV [198]. In vitro investigations have demonstrated that cyclosporine A suppresses SARS and other coronavirus replication [19,199]. In the instance of COVID-19, cyclosporine inhibits the SARS-CoV-2 virus’s cyclophilin function by blocking RdRP and peptidyl-prolyl isomerase activity, thus inhibiting viral replication [200,201,202]. Prospective clinical trials with cyclosporine A have been recently started, (ClinicalTrials.gov ID: NCT04412785). Only cyclosporine, not lopinavir/ritonavir or hydroxychloroquine, was shown to be substantially linked to a decrease in mortality in hospitalized patients [200].

Since ancient times, the benefits of mushrooms, with special reference to medicinal mushrooms’ metabolites in enhancing immune responses and curing infectious diseases, have been investigated by several researchers [201]. The basic immunomodulatory function of bioactive compounds derived from mushrooms is to trigger the immune regulatory system such as lymphocytes, cytotoxic T lymphocytes, T cells, natural killer cells, dendritic cells, and macrophages, leading to the expression and release of cytokines such as tumor necrosis factor-alpha (TNF)-α, interferon-gamma (INF)-γ, and interleukins [201]. Mushroom-derived immunomodulators are divided into four categories: lectins, proteins, polysaccharides, and terpenoids [201]. Lectins are carbohydrate-binding proteins which can be extracted from different taxa of mushrooms. They have certain immune cell activities, such as anticancer and antiproliferative activities [202]. Fungal immunomodulatory proteins are low-molecular-weight proteins (13 kDa and containing 110–114 amino acids) with immunomodulatory properties. They are a type of bioactive compound that can be extracted from edible mushrooms. Further, mushrooms are a valuable source of immunomodulatory polysaccharides [203] with immunomodulatory criteria, such as increasing phagocytic activity and functioning as proinflammatory cytokines [204]. The primary bioactive components of *Ganoderma lucidum* (*G. lucidum*) are polysaccharides and triterpenoids, which have been utilized in folk medicine since ancient times [205]. Polysaccharides from *G. lucidum* have immunomodulatory characteristics, increasing the activity of antigen-presenting cells, the mononuclear phagocyte system, and humoral and cellular immunity. β-Glucans extracted from *G. lucidum* should elicit an immune response via pathogen-associated molecular patterns (PAMPs) [206]. Triterpenoids have antiviral properties via blocking enzymes such as neuraminidase and protease [207,208]. Isolated chemicals from *G. lucidum* might be a viable and promising source in the current SARS-CoV-2 epidemic [205].

Many mushroom species have been extensively studied and recorded as producers of immunomodulators, for example, Amanita pantherina, Agaricus blazei, A. bisporus, Boletus satanas, Cordyceps sinensis, Coprinus cinereus, Ganoderma lucidum, Ischnoderma resinosum, Laetiporus sulphureus, Lactarius deterrimus, Lentinus tigrinus, and Volvariella volvacea (Table S3) [209].

### 6.2. Natural Products from Algae and SARS-CoV-2

Algae are the main part of any body of water, oceans, seas, lakes, rivers, and, as producers [210]. Algae have effectively produced a number of metabolites as natural defense chemicals, allowing them to live in harsh conditions. Many illnesses, including microbial and viral infections, can be treated with algae-derived secondary metabolites and/or chemicals. Furthermore, certain algae species can boost immunity and reduce viral activity in humans. As a result, they may be suggested for usage as a COVID-19 preventative measure [211].

Several marine algae species (seaweeds) contain a large amount of complex bioactive chemicals such as sulphated polysaccharides, which have been found to prevent the reproduction of enveloped viruses, including Nidovirales members. Other rhodophyta components, such as sulphated polysaccharides derived from chlorophyta, such as ulvans, and phaeophyta, such as fucoidans, might be potential antiviral treatment components against SARS-CoV-2 [212,213,214,215,216]. The first potent antiviral action of marine algal polysaccharides was reported by Gerber et al., who described that the polysaccharides extracted from the Rhodophyta (red algae) *Gelidium robustum* protect embryonic eggs against mumps virus or influenza B [210,217]. The exploration of algal components against viral agents after this study carried out by Gerber flourished exponentially [218,219,220]. In 1990, Neushul studied 39 species of marine red algae for their potential as antiviral agents, and he found 36 tested seaweed extracts had positive effects against viral infection [221]. His study revealed three compounds that are nonreactive and seven mildly reactive extracts, while 29 algal species collected from central and southern California showed an active response against viral infections [221].

Dieckol (C36H22O18) is a phlorotannin isolated from the brown alga *Ecklonia cava* that has been shown to have the highest effective SARS-CoV 3CLpro trans-/cis-cleavage inhibitory action in a dose-dependent and competitive way with no cytotoxicity [222]. Griffithsin, a lectin derived from the red algae Griffithsia spp, binds to oligosaccharides on the surfaces of different viral spike proteins and possesses antiviral properties against SARS-CoV [212] and MERS-CoV [223]. Griffithsin suppresses a wide variety of Coronaviridae *in vitro*, including HCoV-229E, HCoV-OC43, and HCoV-NL63, and in vivo activity against SARS-CoV-1 in a mouse model system after intranasal treatment [86]. Because griffithsin inhibits viral entry, reverse-transcriptase activity, integrase activity, and protease activity, it may be helpful against SARS-CoV-2 [224,225].

Carrageenan (C23H23FN4O7Zn), a sulfated polysaccharide derived from the red edible seaweed *Chondrus crispus*, has shown activity against SARS-CoV-2 by inhibiting its replication in vitro [226,227]. Carrageenans are proven to act against nonenveloped and enveloped viruses by inhibiting their binding to host cells during the initial stages of infections [228,229]. Combinations of carrageenan and griffithsin have shown synergistic efficacy, especially against current SARS-CoV-2 mutations [230].

Spirulina, derived from *Arthrospira platensis*, is a widely viable dietary supplement that is high in phenolic acids, necessary fatty acids, sulfated polysaccharides, and vitamin B12. It is a kind of nutritious blue–green algae rich in phenolic acids, essential fatty acids, sulfated polysaccharides, and vitamin B12 [231]. By attaching to the S1 motif of 36 spikes and inhibiting the contact of spikes with their receptor, it exhibits significant antiviral activity against pseudotype coronaviruses [211]. Additionally, calcium spirulan exhibits potential antiviral activities against herpes simplex 1 (HSV-1), measles, mumps, influenza, polio, Coxsackie, human immunodeficiency (HIV), and human cytomegalo (HCMV) [232].

Galactan sulphate extracted from the marine red algae *Aghardhiella tenera* showed activity against the viral infections HIV-1 and HIV-2, and it was proven to be an active antiviral agent [233]. Cyanovirin-N (CV-N), purified from the cyanobacterium *Nostoc ellipsosporum*, is a cyanobacterial protein that has strong antiviral action toward HIV [234]. CV-N has been demonstrated to have a strong affinity for HIV gp120 and to inhibit the envelope glycoprotein-mediated membrane fusion process involved with HIV-1 entrance. CV-N exhibits wide antiviral efficacy against a variety of enveloped viruses and many stages in the HIV entry process [235].

Naviculan, a sulfated polysaccharide produced from the diatom species *Navicula directa*, has potential antiviral activity against HSV-1 and HSV-2 [236]. Fucoidan is also a sulfated polysaccharide isolated from the brown seaweed *Fucus vesiculosus* [237]. Fucoidan is well-known for its antioxidants, anti-inflammatory [238], antidiabetic [239], anticoagulant [240], and antiviral [241] properties. Fucoidan might be a promising choice for treating a wide variety of COVID-19 patients [242]. Different antiviral agents derived from marine algae are presented in (Table S4).

### 6.3. Antiviral Peptides Derived from Scorpion Venoms

Scorpions (over 2400 described species) are particularly fascinating for the potency of their venom, which is used to disrupt biochemical and physiological processes in target organisms. Scorpion venom has proven to be a rich source of bioactive molecules, especially ion channels blockers. In the recent years, it has been increasingly recognized that scorpion venoms also have an abundant supply of AMPs [26], including antiviral peptides [243,244]. The evolutionary success of scorpions can be associated, in part, with their relatively simple but highly effective innate immune system including venom AMPs. Their effectiveness relies primarily in the recognition of infectious organisms and consequent activation of cellular and humoral responses leading to the clearance of foreign invaders.

The crude venom of various scorpions and their purified toxins revealed antiviral activities in vivo and in vitro and are considered as a rich source for developing potential antiviral drugs [245]. Li and his co-workers (2011) identified the scorpion venom antimicrobial peptide of mucroporin-M1 (17-amino acids; LFRLIKSLIKRLVSAFK) from *Lychas mucronatus*. Mucroporin-M1 showed viricidal activity against measles virus (MeV propagated in Vero cell monolayers) (EC50 3.52 µM) through binding directly with the virus particles (virus envelope), thereby diminishing the virus infectivity. Mucroporin-M1 exhibited about 20% repression of MeV infection within 0–12 h post treatment, and no observable repression activity was detected after 12 hrs. When mucroporin-M1 was mixed with MeV directly and incubated for 1 h before infecting cells, it showed approximately 100% inhibition. In addition, mucroporin-M1 revealed virucidal activity against SARS-CoV (EC50 7.12 µM) and influenza H5N1 viruses (EC50 1.03 µM). Moreover, the activity of mucroporin-M1 on hepatitis B virus (HBV) has been examined using both in vitro and in vivo studies [246]. Mucroporin-M1 inhibited the replication of HBV through the selective activation of mitogen-activated protein kinase (MAPK) pathway, which led to the inhibition of HNF4α expression and consequently decreased the binding of HNF4α to the HBV promoter [246].

From the venom of the Egyptian scorpion *Scorpio maurus palmatus*, the scorpine-like peptide of Smp76 (8398.0 Da, 76 amino acids) was isolated using proteotranscriptomic analyses [29] and showed potent antiviral activity against HCV and dengue virus (DENV) [244]. Using in vitro studies, the native Smp76 blocked the early stages of HCV and DENV life cycles through binding with viral particles (without affecting the replication of both viruses). On the other hand, the group of Zhijian Cao examined the effect of recombinant Smp76 (rSmp76 expressed in Escherichia coli BL21) on DENV and Zika virus (ZIKV) [247]. The rSmp76 significantly inhibited the viral infection of both DENV and ZIKV (propagated in primary mouse macrophages and cultured cell lines) through enhancing the expression of type-I interferon (type-I IFN). Thus, this investigation revealed that there is no direct binding between rSmp76 and virus particles. In addition, the group of Zhijian Cao [88] revealed the inhibitory efficacy (in a dose-dependent manner at noncytotoxic concentrations) and the molecular mechanism of the recombinant rEv37 (purified from the scorpion venom *Euscorpiops validus* on various viruses including DENV-2, HCV, ZIKV, and herpes simplex virus type 1 (HSV-1). The mechanistic studies revealed that Ev37 is preventing low pH-dependent fusion of the viral membrane-endosomal membrane through alkalizing the acidic organelles.

Hp1036 and Hp1239 are two short venom cationic peptides (13 amino acids) derived from the scorpion *Heterometrus petersii* with a potent inhibitory effect on HSV-1 (HSV-1 propagated in Vero cells; EC50 = 0.43 and 0.41 µM, respectively) infection in vitro [245]. The virucidal activities of both peptides mainly depend on the ability of these toxins (i) to induce morphological changes of HSV-1 virion and (ii) to inhibit viral attachment and entry steps. Like Hp1036 and Hp1239, several antiviral peptides have been identified from scorpion venoms such as Hp1090 [248,249], Ctry2459 [248,250], and Kn2-7 [248,251]. These peptides showed similar virucidal activities (as Hp1036 and Hp1239) against enveloped virus. However, they have no inhibitory effects on the replication of viruses when the toxins are added to the cells after infection. Recently, the scorpion defensin of BmKDfsin3 has been identified from the venom of *Mesobuthus martensii Karsch*. BmKDfsin3 effectively inhibited the infection of HCV (IC50 3.35 µM) through the alteration of the viral attachment and HCV life cycle (via the suppression of the p38 MAPK pathway) within Huh7.5.1 cells [243]. As a molecular probe or tool, BmKDfsin3 indicated that HCV infection was closely associated with p38 activation, and the p38 MAPK signal pathway may play an important role during COVID-19 infection. Based on this finding, Dr. Grimes’s group revealed the link between upregulation of p38 activity and SARS-CoV-2, and they proposed that disproportionately upregulated p38 expression may promote the inflammatory response in the COVID-19 infection, which was explained by two pathways. First, during SARS-CoV-2 viral entry, (ACE2) activity is impaired. ACE2 is strongly expressed in the lungs and heart and transforms angiotensin II into angiotensin 1–7. Angiotensin II signals proinflammatory, prothrombotic, and provasoconstrictive activity via p38 MAPK stimulation, which is combated by angiotensin 1–7 downregulation of p38 activity. When ACE2 is lost during viral entrance, it may shift the balance to damaging p38 signaling via angiotensin II. Second, like other RNA respiratory viruses that may mimic p38 activity to enhance replication, SARS-CoV has been shown to effectively upregulate p38 activity via a viral protein. Because SARS-CoV and SARS-CoV-2 are so similar, the latter may use a similar pathway. Thus, SARS-CoV-2 could cause severe inflammation by activating p38 and downregulating a key inhibitory pathway, while at the same time, taking advantage of p38 activity to replicate. The therapeutic blocking of p38 could consequently impair COVID-19 infection. Thus, they revealed a common pattern that virus-induced p38 MAPK activation facilitates viral infection, and targeting p38 MAPK is a promising antiviral strategy [252]. It is a successful example to use the knowledge of animal toxic peptide for probing and addressing some of the major health challenges of our time such as viral infection. Accordingly, the scorpion venom AMPs revealed potent antiviral activity and working through various mechanisms as (i) inhibit viral infectivity by their direct damage of the viral envelop; (ii) block viral entry steps through suppression the attachment of viral glycoproteins with cell receptors or preventing low pH-dependent fusion; and (iii) inhibit viral replication inside host cells and stop viral infection to other cells.

### 6.4. Natural Compounds from Plants and SARS-CoV-2

Several natural products derived from plants have been identified based on in silico screening and in vitro studies as promising inhibitors against SARS-CoV-2 (Table 7).

Artemisinins are a class of artemisinin-related medicines that demonstrated a variety of pharmacological actions, including anticancer, antiviral, and immunological regulation. Cao et al. reported the antiviral activity of nine artemisinin-related compounds against SARS-CoV-2 and further investigated their mechanism of action but also predicted their pharmacokinetic properties [253]. The results showed that arteannuin B, among the tested artemisinin compounds, displayed the most potent antiviral activity against SARS-CoV-2 with an EC_50_ of 10 µM, while artesunate and dihydroartemisinin exhibited identical EC_50_ values of 13 µM. Interestingly, although lumefantrine demonstrated a low EC_50_ of 23 µM, the results indicated a high therapeutic potential. Furthermore, arteannuin B and lumefantrine showed to be functioned in the postentry stage of SARS-CoV-2 infection [253]. Catechins, also known as flavan-3-ol, are potent antioxidants, anti-inflammatory, and antiviral agents that have been approved for human use. Interestingly, the treatment of ten swab-positive SARS-CoV-2 patients with catechins (total catechins: 840 mg; total EGCG: 595 mg) was demonstrated at a median of 9 days, with a range of 7–15 days, in which all patients completely healed and exhibited no symptoms. While, at a median of 9 days, seven patients transitioned to a negative SARS-CoV-2 nasopharyngeal swab test, one-third of the patients who remained swab-positive showed a significant reduction in infection. Notable, inflammation markers such as –1 antitrypsin, C-reactive protein, and eosinophils were also considerably dropped [254].

Baicalin and baicalein are key ingredients of plant species in the Scutellaria genus and are the source of *Scutellaria baicalensis’* medicinal characteristics [255]. Zandi et al. showed that baicalein and baicalin demonstrated substantial antiviral efficacy against SARS-CoV-2 by potentially inhibiting SARS-CoV-2 RdRp [256]. The results showed that baicalein possesses a higher inhibitory activity than both baicalin and glycyrrhizin [257]. Cocrystallization investigations showed that baicalein binds specifically and uniquely to the SARS-CoV-2 RdRp; therefore, it is considered as a selective and potent non-nucleoside polymerase inhibitor. On the other hand, baicalin showed a considerable inhibitory activity against SARS-CoV-2 3CL-Pro activity [258]. Kumar et al. investigated the inhibitory activity of ten essential oils against ACE2 activity [259]. Geranium and lemon oils demonstrated potent ACE2 inhibitory activity in epithelial cells based on immunoblotting and qPCR analysis. Gas chromatography-mass spectrometry (GC-MS) analysis showed that citronellol, geraniol, and neryl acetate were the main constituents of geranium oil, whereas limonene was the main constituent of lemon oil. Among the identified constituents, citronellol and limonene substantially attenuated ACE2 expression in epithelial cells. These findings indicate that geranium and lemon essential oils, as well as their derivative chemicals, may be considered as natural antiviral agents against SARS-CoV-2 [259].

In an interesting study, Xiao et al. performed molecular modeling and in vitro screening for a set of 15 natural compounds against SARS-CoV-2 which led to the identification of myricetin as a potent inhibitor against SARS-CoV-2 with an IC_50_ of 3.7 µM [260]. Detailed in silico molecular docking study revealed that myricetin has a high binding affinity toward SARS-CoV-2 Mpro protein. In the binding pocket of SARS-CoV-2 Mpro, the chromone ring of myricetin hydrophobically interacts with His41 residue, while the 3′, 4′, and 7-hydroxyl groups form hydrogen bonds with Phe140, Glu166, and Asp187 residues. Noticeably, myricetin demonstrated a considerable impact on bleomycin-induced pulmonary inflammation by reducing inflammatory cell infiltration and the production of inflammatory cytokines (IL-6, IL-1 α, TNF- α, and IFN- γ) [260].

Biflavonoids (amentoflavone, bilobetin, ginkgetin, and sciadopitysin) extracted from *Torreya nucifera* displayed a considerable inhibitory activity against SARS-CoV-2 3CLpro activity (IC_50_ 8.3-72.3 µM). Among different bioflavonoids, amentoflavone showed the most potent inhibitory efficacy, with IC_50_ of 8.3 µM [261]. Based on these results, a selection of natural compounds including amentoflavone, dieckol, hirsutenone, cryptotanshinone, xanthoangelol E, tomentin E, psoralidin, scutellarein, myricetin, and caffeic acid have been considered as potent inhibitors against SARS-CoV-2. However, most of these compounds are phenolic molecules that are characterized by low bioavailability and fast elimination, which might limit their therapeutic application [262]. In an interesting study by Zhuang et al., the Cinnamomi cortex extract demonstrated a moderate inhibitory activity against wild-type coronavirus (wtSARS-CoV) and SARS-CoV S pseudovirus [263]. Further investigations showed that the extract of cinnamon-derived procyanidin type A possesses a high binding affinity to the ACE2 receptor as well as glycans on the viral spike protein, which may lead to a significant block of SARS-CoV2 infection [264].

Herbacetin, rhoifolin, and pectolinarin have been identified as potent inhibitors for SARS-CoV 3CLpro enzymatic activity during screening a library of flavonoids (IC_50_ 25–38 µM). A tryptophan-based fluorescence assay and induced-fit docking analysis revealed that the three flavonoids interact and bind through their sugar moieties to the S1, S2, and S30 subunits of SARS-CoV 3CLpro. Accordingly, herbacetin, rhoifolin, and pectolinarin have been considered as templates for designing functionally enhanced and potent inhibitors for SARS-CoV-2 activity [265]. Chlorinated isoxazole maslinic acid has been identified as a potent antiviral compound against SARS-CoV-2 major protease based on an in silico molecular docking screening of seventeen natural maslinic and oleanolic acids analogues. In further investigation, chlorinated isoxazole maslinic acid demonstrated in vitro potential antiviral activity against SARS-CoV-2 viral proliferation [266]. Wen et al. investigated the antiviral activity of a set of 221 natural plant metabolites, including terpene and lignin derivatives, against SARS-CoV [267]. Further in silico molecular modeling the investigation of the inhibitory effect of the best 20 compounds demonstrated that abietane-type diterpenoids and lignoids exhibit a high binding affinity toward SARS-CoV 3CLpro [267]. Naringenin, a flavanone present in grapes, has been shown to reduce hypertensive renovascular destruction in vivo by lowering expression of ACE2 [268]. These findings suggest that flavonoids may be useful in preventing SARS-CoV-2 infection via modulating ACE2 receptor [269].

On the other hand, various plant extracts have been investigated and screened for their antiviral activity against SARS-CoV. The crude extracts of the African plant Trifolium species (Fabaceae) prevent the entry of SARS-CoV [270]. Plant extracts containing compounds such as saponins, polyphenols, flavonoids, terpenes, alkaloids, coumarins and chalcones, anthraquinones, polysaccharides, and glycoproteins exhibited potential inhibitory activity against the viral replication and/or entry [271]. Moreover, saponins displayed the ability to block SARS-CoV entry and penetration into Vero cell line [272].

Lectins are carbohydrate-binding proteins found in a variety of plants, including tobacco, soy, and leeks, that can act as immunologic receptors and defense proteins. The antiviral and therapeutic effects of lectins depend on the binding affinity of lectin to the sugar moiety. The purified Mannose binding lectin from *Hippeastrum hybrid* (Amaryllidaceae) and N-acetyl glucosamine-specific lectins from *Urtica dioica* L., (Urticaceae) and Nicotiana tabacum exhibited the antiviral activity against SARS-CoV by preventing virus binding to the ACE-2 receptor and blocking virus entry [273,274]. Several studies have shown that essential oils from medicinal plants, including *Citrus* spp., *Hyssopus officinalis*, *Illicium* spp., mayweeds, tea trees, *Mentha* spp., *Santalum* spp., *Pinus* spp., and certain other aromatic plants, have antiviral properties [258]. Essential oils have the ability to penetrate the lipid bilayer layer of the viral envelope in an abrupt manner, altering the fluidity of the membrane [275]. Because of their lipophilic character, monoterpenes, oxygenated sesquiterpenes, and phenylpropanoids in essential oils can disturb the phospholipid bilayer barrier of human coronaviruses, interfering with the shape of the proteins of enveloped virus throughout the infection [276]. Several other natural products are summarized in (Table 7).

### 6.5. Antiviral Natural Products under Clinical Investigations against SARS-CoV-2

Recently, several natural products have been clinically explored as potential drugs for the COVID-19 disease (Table 8). Artemisinin and artesunate obtained from *Artemisia* spp were explored in nine clinical trials for the treatment of COVID-19 (ClinicalTrials.gov, 2020). Both artemisinin and artesunate are used in the initial treatment of severe malaria. Artemisinin is a sesquiterpene lactone extracted from *Artemisia annua*, while artesunate is an artemisinin derivative, a sesquiterpenoid, a dicarboxylic acid monoester, a cyclic acetal, a semisynthetic derivative, and a hemisuccinate. Out of them, six were registered in the United States National Library of Medicine, while the other three appeared in the Chinese clinical trial registry (ChiCTR 2020). In the clinical study, Artemisia was used as tea (225 mg/1350 mg) per day, oral (one 8 oz brewed tea (two bags) three times a day) from days 1–14. The evaluation of the safety and efficacies on morbidity of COVID-19 patients showed a decrease in the course of the disease and viral load in symptomatic stable positive swab COVID-19 patients (ClinicalTrials.gov 2020).

Berberine is a nonbasic and quaternary benzylisoquinoline alkaloid, which is mainly produced by the genus Berberis, which has 450–500 species in the Berberidaceae family. Recently, Hosseinzadeh investigated the clinical impact of berberine on 40 COVID-19 patients (IRCT registration number: IRCT20081019001369N2). The preliminary results indicated that berberine improved lymphopenia and reduced clinical symptoms (fever, cough, and myalgia) (https://en.irct.ir/trial/46868, accessed on 7 April 2020).

Colchicine is an alkaloid that is found naturally in *Colchicum autumnale* (Family Liliaceae), as well as *Gloriosa superba*. Salehzadeh et al. reported the clinical effect of colchicine on 100 COVID-19 patients. Colchicine was found to be helpful in reducing systemic symptoms of COVID-19 by suppressing inflammatory biomarkers [291].

Quercetin is a flavonoid derivative found in various fruits and vegetables, including *Foeniculum vulgare* and *Allium cepa*. It has been reported that quercetin potentially blocks SARS-CoV protease (82%) [292]. Based on in silico molecular modeling studies, quercetin showed a high binding affinity toward SARS-CoV-2 3CLpro and other crucial targets [293]. Di Pierro et al. recently showed that the treatment of patients, with mild COVID-19 severity, with the bioavailable form of quercetin, Quercetin Phytosome^®^, may have a milder symptomatology, a shorter time to virus clearance, and higher probabilities of a benign earlier resolution of the disease. Indeed, after 1 week of Quercetin Phytosome treatment, 16 patients tested negative for SARS-CoV-2, and 12 patients had SARS-CoV-2 symptoms diminished [294].

Emodin is an anthraquinone derivative extracted from the roots and barks of *Rheum palmatum, Polygonum cuspidatum,* and *Polygonum multiflorum*. It is also produced by several fungi species and Chinese herbs. Emodin demonstrated a potential ability to inhibit the SARS-CoV by targeting ACE2 receptor [279]. Recently, Jang et al. identified emodin as a potent inhibitor of SARS-CoV-2 activity in human Calu-3 lung cells based on a virtual screening of 6218 drugs and cell-based assays [295].

Resveratrol is a phytoalexin generated by several plants under stress-inducing conditions and is presented in many dietaries including berries and grapes, their juices, and wines. Resveratrol demonstrated the potential effects on several factors associated with cardiovascular diseases; therefore, it has been highlighted as a functional supplement to reduce severity of COVID-19 in patients with cardiovascular complications. Gligorijević et al. reported the impact of resveratrol in mediating cardiovascular disorders and cardiovascular complications, which are related to COVID-19 severity. Resveratrol showed the ability to attenuate several pathways involved in SARS-CoV-2 pathogenesis, including the downregulation of cytokine release and expression of ACE2 [296].

Tetrandrine (Hanfangchin A) is a bisbenzylisoquinoline extracted from the perennial vine plant *Stephania tetrandra* (Chinese patent WO2004009106A1, 2002) and has been used in the traditional Chinese medicine [297]. The clinical application of tetrandrine in the treatment of COVID-19 (TT-NPC) has been submitted in March 2020 (https://www.clinicaltrials.gov/ct2/history/NCT04308317?V_1=View, accessed on 13 March 2020). The results showed that the combination of tetrandrine with standard treatment regimens improved prognosis, reduced the clinical progress, and reduced the pulmonary fibrosis in patients with mild and severe neocoronary pneumonia.

Glycyrrhizin is the main saponin in licorice that is considered as an analogue of the pentacyclic triterpene oleanane. Glycyrrhizin displayed a wide range of medicinal benefits including anti-inflammatory, antiulcer, antiallergic, hepatoprotective, and antiviral [298,299]. Van de Sand et al. reported the ability of glycyrrhizin to potentially in vitro inhibit SARS-CoV-2 replication [300]. In further studies, Ding et al. reported that clinical treatment of patients with a derivative of glycyrrhetinic acid and diammonium glycyrrhizinate helped in the recovery from severe COVID-19 symptoms. Based on these results, the authors suggested that mixing vitamin C with diammonium glycyrrhizinate could be a promising strategy for the treatment of severe COVID-19 symptoms [301]. Further, several studies endorsed the definite clinical effect of traditional Chinese medicine (TCM), in which the glycyrrhizin or licorice extract is used, for COVID-19 treatment [302].

Ursodeoxycholic acid (UDCA) is a hydrophilic epimer of chenodeoxycholate that demonstrated the ability to stimulate alveolar fluid clearance in lipopolysaccharide-induced pulmonary edema, leading to an improvement of acute respiratory distress syndrome. These findings indicate that UDCA may have potential therapeutic effects on COVID-19-induced pneumonia and related lung oedema through tackling cytokine storm syndrome [303].

## 7. Conclusions

In this review, we summarized the latest updates regarding coronaviruses, with special focus on SARS-CoV-2. The detailed molecular structures of SARS-CoV-2 proteins were also discussed, which helped in the discovery of many drugs against COVID-19. Many vaccines have been developed recently for SARS-CoV-2. Nevertheless, the global health concerns about this pandemic are expected to go on for at least another 10 years. Scientists are struggling to fully understand the potency and the protection period of the developed vaccines which would require a comprehensive research and trials. Further clinical studies should be conducted to develop a new vaccine which would be applicable for children below 12 years age and pregnant and lactating females. This would certainly depend on shedding the lifecycle of the virus and interactions between the virus and host cell. However, this became extremely challenge after the discovery of the possibility of the virus to mutate into several other mutated forms. Accordingly, several alternative approaches are urgently needed to be established to attenuate the virus replications. Natural products derived from the algae, fungal, and plant kingdoms were concluded to be able to regulate the production and release of proinflammatory cytokines, interfere with the virus’s development in host cells, and modify certain molecular pathways. These natural compounds might be used as food supplements (such as reishi mushroom) and/or in COVID-19 therapy. However, patients should be aware that using a supplement containing one of these natural chemicals to prevent COVID-19 or treat the infection is still not suggested without a direct supervision of a specialist. A clinician’s recommendation is to provide these natural medicines to patients with caution, even if they are healthy. Since several natural agents against COVID-19 are already in preclinical and clinical trials, investigating the effect of these drugs against the virus replication would be a promising strategy to find novel antiviral agents against SARS-CoV-2. This approach is definitely viable and has translational prospective and deserves immediate consideration by the global scientific community.

## Figures and Tables

**Table 1:** The known 3D structures of main protease available on protein data bank (PDB).

PDB ID	Resolution	Macromolecule	Ligand	Reference
*6lU7*	2.16	- SARS-CoV-2 main protease - synthetic construct	N3 inhibitor	[95]
*6M2N*	2.20	- 3CLpro - SARS-CoV-2 main protease	Baicalein	[96]
*7K3T*	1.2	- 3C-like protease (3CL pro)	-	
*6YB7*	1.25	- SARS-CoV-2 main protease - ORF1ab polyprotein	-	
*7JKV*	1.25	-3CLpro	N-[(2S)-1-({(1S,2S)-1-(1,3-benzothiazol-2-yl)-1-hydroxy-3-[(3S)-2-oxopyrrolidin-3-yl]propan-2-yl}amino)-4-methyl-1-oxopentan-2-yl]-4-methoxy-1H-indole-2-carboxamide	[97]
*5R8T*	1.27	- 3CLpro - SARS-CoV-2 main protease	-	[98]
*6XKH*	1.28	- 3CLpro	-	
*5R82*	1.31	- 3CLpro - SARS-CoV-2 main protease.	6-(ethylamino)pyridine-3-carbonitrile	
*5RH4*	1.34	- 3CLpro - SARS-CoV-2 main protease	(2R)-2-(6-chloro-9H-carbazol-2-yl)propanoic acid	
*5RGJ*	1.34	- 3CLpro - SARS-CoV-2 main protease	(5S)-7-(pyrazin-2-yl)-2-oxa-7-azaspiro[4 .4]nonane	[98]
*7K40*	1.35	- 3CLpro	Boceprevir (bound form)	
*6XHN*	1.377	- 3CLpro	(3S)-3-{[N-(4-methoxy-1H-indole-2-carbonyl)-L-leucyl]amino}-2-oxo-4-[(3S)-2-oxopyrrolidin-3-yl]butyl 2-cyanobenzoate	[99]
*7BRR*	1.4	- 3C-like proteinase - 3CL-PRO - 3CLp	(1S,2S)-2-({N-[(benzyloxy)carbonyl]-L-leucyl}amino)-1-hydroxy-3-[(3S)-2-oxopyrrolidin-3-yl]propane-1-sulfonic acid	[100]
*6XHM*	1.41	- 3CLpro	N-[(2S)-1-({(2S)-4-hydroxy-3-oxo-1-[(3S)-2-oxopyrrolidin-3-yl]butan-2-yl}amino)-4-methyl-1-oxopentan-2-yl]-4-methoxy-1H-indole-2-carboxamide	[99]
*5RFD*	1.41	- 3CLpro - SARS-CoV-2 main protease	2-[(methylsulfonyl)methyl]-1H-benzimidazole	[98]
*5RGR*	1.41	- 3CLpro - SARS-CoV-2 main protease	N,1-dimethyl-N-(propan-2-yl)-1H-pyrazolo[3,4-d]pyrimidin-4-amine	[98]
*5RF9*	1.43	- 3CLpro - SARS-CoV-2 main protease	1-[(2~{S})-2-methylmorpholin-4-yl]-2-pyrazol-1-yl-ethanone	[98]
*5RFW*	1.43	- 3CLpro - SARS-CoV-2 main protease	1-{4-[(thiophen-2-yl)methyl]piperazin-1-yl}ethan-1-one	[98]
*5RHB*	1.43	- 3CLpro - SARS-CoV-2 main protease	(E)-1-(pyrimidin-2-yl)methanimine	[98]
*5RGW*	1.43	- 3CLpro - SARS-CoV-2 main protease	2-(5-cyanopyridin-3-yl)-N-(pyridin-3-yl)acetamide	
*5RF8*	1.44	- 3CLpro - SARS-CoV-2 main protease	4-amino-N-(pyridin-2-yl)benzenesulfonamide	[98]
*6WNP*	1.443	-3CLpro - ORF1ab polyprotein	Boceprevir (bound form)	
*6XHO*	1.446	- 3CLpro	Ethyl (2E,4S)-4-{[N-(4-methoxy-1H-indole-2-carbonyl)-L-leucyl]amino}-5-[(3S)-2-oxopyrrolidin-3-yl]pent-2-enoate	[99]
*5RF6*	1.45	- 3CLpro - SARS-CoV-2 main protease	5-(1,4-oxazepan-4-yl)pyridine-2-carbonitrile	[98]
*6XR3*	1.45	- 3CLpro	N-[(2S)-1-({(1S,2S)-1-(1,3-benzothiazol-2-yl)-1-hydroxy-3-[(3S)-2-oxopyrrolidin-3-yl]propan-2-yl}amino)-4-methyl-1-oxopentan-2-yl]-4-methoxy-1H-indole-2-carboxamide	
*6XBG*	1.45	- SARS-CoV-2 main protease - 3CLpro	UAW246 inhibitor	[101]
*6W79*	1.46	- SARS-CoV-2 main protease	N-(4-tert-butylphenyl)-N-[(1R)-2-(cyclohexylamino)-2-oxo-1-(pyridin-3-yl)ethyl]-1H-imidazole-4-carboxamide	
*5RFE*	1.46	- 3CLpro - SARS-CoV-2 main protease	N-[(4-cyanophenyl)methyl]morpholine-4-carboxamide	[98]
*5RED*	1.47	- 3CLpro - SARS-CoV-2 main protease	4-[2-(phenylsulfanyl)ethyl]morpholine	[98]
*6XHL*	1.471	- 3CLpro	N-[(2S)-1-({(2S)-4-hydroxy-3-oxo-1-[(3S)-2-oxopyrrolidin-3-yl]butan-2-yl}amino)-4-methyl-1-oxopentan-2-yl]-4-methoxy-1H-indole-2-carboxamide	[99]
*6WCO*	1.48	- SARS-CoV-2 main protease	N-(4-tert-butylphenyl)-N-[(1R)-2-(cyclopentylamino)-2-oxo-1-(pyridin-3-yl)ethyl]-1H-imidazole-4-carboxamide	
*5RFB*	1.48	- 3CLpro - SARS-CoV-2 main protease	N-[(1-methyl-1H-1,2,3-triazol-4-yl)methyl]ethanamine	[98]
*5RFV*	1.48	- 3CLpro - SARS-CoV-2 main protease	1-[4-(thiophene-2-carbonyl)piperazin-1-yl]ethan-1-one	[98]
*5C5O*	1.5	- 3CLpro	(2S)-3-(1H-imidazol-5-yl)-2-({[(3S,4aR,8aS)-2-(N-phenyl-beta-alanyl)decahydroisoquinolin-3-yl]methyl}amino)propanal	
*5RF3*	1.5	- 3CLpro - SARS-CoV-2 main protease.	pyrimidin-5-amine	[98]
*5RGK*	1.43	- 3CLpro - SARS-CoV-2 main protease	2-fluoro-N-[2-(pyridin-4-yl)ethyl]benzamide	[98]

**Table 2:** The known 3D structures of Papain-like protease (PLpro) available on protein data bank (PDB).

PDB ID	Resolution	Source Organism	Macromolecule	Ligand	Reference
*7CMD*	2.59	- SARS-CoV-2	- PLpro	GRL0617	[107]
*6WX4*	1.655	- SARS-CoV-2 - Saltans group	- Nonstructural protein 3 - PLpro - pp1ab - ORF1ab polyprotein	VIR251	[110]
*6WUU*	2.79	- SARS-CoV-2 - Synthetic construct	- PLpro - pp1ab - ORF1ab polyprotein	VIR250	[110]
*6YVA*	3.18	- SARS-CoV-2 - Mus musculus	- Replicase polyprotein 1a - pp1ab - ORF1ab polyprotein - Ubiquitin-like protein ISG15 - Interferon-induced 15 kDa protein - Interferon-induced 17 kDa protein - IP17 - Ubiquitin cross-reactive protein	mISG15	[40]
*7JRN*	2.48	- SARS-CoV-2	- PLpro	5-amino-2-methyl-N-[(1R)-1-naphthalen-1-ylethyl]benzamide	-
*6W9C*	2.7	- SARS-CoV-2	- Papain-like proteinase		-
*7CJM*	3.2	- SARS-CoV-2	- Nonstructural protein 3 - PLpro	5-amino-2-methyl-N-[(1R)-1-naphthalen-1-ylethyl]benzamide	-
*6XA9*	2.9	- SARS-CoV-2 - Homo sapiens	- PLpro - Nonstructural - CTD-propargylamide - Interferon-induced 15 kDa protein - Interferon-induced 17 kDa protein - IP17 - Ubiquitin cross-reactive protein - hUCRP ein 3	ISG15	-
*7CMD*	2.59	- SARS-CoV-2	- Replicase polyprotein 1ab - pp1ab - ORF1ab polyprotein	5-amino-2-methyl-N-[(1R)-1-naphthalen-1-ylethyl]benzamide	[108]
*6XAA*	2.7	- SARS-CoV-2	- PLpro - Nonstructural protein 3 - Ubiquitin-propargylamide	ubiquitin propargylamide	-
*7CJD*	2.501	- SARS-CoV-2	- Replicase polyprotein 1ab	-	[108]

**Table 3:** The known 3D structures of Spike glycoprotein available on protein data bank (PDB).

PDB ID	Resolution	Source Organism	Macromolecule	Reference
*6M1V*	1.5	- SARS-CoV-2	- spike protein	-
*7JMP*	1.712	- SARS-CoV-2 - Homo sapiens	- Spike protein S1 - COVA2-39 heavy chain - COVA2-39 light chain	-
*6YZ5*	1.8	- SARS-CoV-2 - Lama glama	- Spike glycoprotein - Nanobody H11-D4	-
*7BZ5*	1.84	- SARS-CoV-2 - Homo sapiens	- Spike protein S1 - Heavy chain of B38 - Light chain of B38	[115]
*6ZBP*	1.85	- SARS-CoV-2 - Lama glama	- Spike glycoprotein - H11-H4	
*7C8V*	2.15	- Synthetic construct - SARS-CoV-2	- Synthetic nanobody SR4 - Spike glycoprotein	-
*6WAQ*	2.2	- Lama glama - SARS-CoV	- nanobody SARS VHH-72 - Spike glycoprotein	-
*6XC4*	2.341	- SARS-CoV-2	- Spike protein S1 - CC12.3 heavy chain - CC12.3 light chain	[34]
*7JMO*	2.359	- SARS-CoV-2 - Homo sapiens	- Spike protein S1 - COVA2-04 heavy chain - COVA2-04 light chain	
*6XLU*	2.4	- SARS-CoV-2	- Spike glycoprotein	[15]
*7CHB*	2.4	- Homo sapiens - SARS-CoV-2	- BD-236 Fab heavy chain - BD-236 Fab light chain - SARS-CoV-2 receptor binding domain	-
*6YLA*	2.42	- SARS-CoV-2 - Homo sapiens	- Spike glycoprotein - Heavy Chain - Light chain	-
*6M0J*	2.45	- Homo sapiens - SARS-CoV-2	- Angiotensin-converting enzyme 2 - Spike receptor binding domain	[116]
*6VYB*	3.20	- Homo sapiens - SARS-CoV-2	- Spike glycoprotein	[116]

**Table 4:** The known 3D structures of RNA-dependent RNA polymerase (RdRp) available on protein data bank (PDB).

PDB ID	Resolution	Source Organism	Macromolecule Name	Reference
*7BTF*	2.95	- SARS-CoV-2	- NSP12 - pp1ab - ORF1ab polyprotein	[108]
		- SARS-CoV-2	- NSP7 - pp1ab - ORF1ab polyprotein	
		- SARS-CoV-2	- NSP8 - pp1ab - ORF1ab polyprotein	
*6M71*	2.9	- SARS-CoV-2	- NSP 12 - pp1ab - ORF1ab polyprotein	[108]
		- SARS-CoV-2	- NSP 7 - pp1ab - ORF1ab polyprotein	
		- SARS-CoV-2	- NSP 8 - pp1ab - ORF1ab polyprotein	
*7BZF*	3.26	- SARS-CoV-2	- NSP 8-1	[72,102]
		- SARS-CoV-2	- NSP7	
		- SARS-CoV-2	- RNA-directed RNA polymerase - NSP12	
*7C2K*	2.93	- SARS-CoV-2	- RNA-directed RNA polymerase - NSP12	[102]
		- SARS-CoV-2	- NSP 8-1	
		- SARS-CoV-2	- NSP7	
*6YYT*	2.90	- SARS-CoV-2 - Synthetic construct	- NSP12	[117]
		- SARS-CoV-2 - Synthetic construct	- NSP 8	
		- SARS-CoV-2 - Synthetic construct	- NSP7	

**Table 5:** The known 3D structures of nucleocapsid available on protein data bank (PDB).

PDB ID	Resolution	Source of Organism	Macromolecules Name	Reference
*6M3M*	2.70	- SARS-CoV-2	- Nucleoprotein	[123]
*6WZO*	1.42	- SARS-CoV-2	- Nucleoprotein - Nucleocapsid protein - Protein N	[123]
*6WZQ*	1.45	- SARS-CoV-2	- Nucleoprotein - Nucleocapsid protein - Protein N	[123]
*6M3M*	2.7	- SARS-CoV-2	- Nucleocapsid protein	[122]
*6WKP*	2.67	- SARS-CoV-2	- Nucleocapsid protein - Nucleoprotein - Protein N	-
*6YUN*	1.44	- SARS-CoV-2	- Nucleoprotein - Nucleocapsid protein - Protein N	-
*7CE0*	1.5	- SARS-CoV-2	- Nucleoprotein - Nucleocapsid protein - Protein N	-
*7CDZ*	1.8	- SARS-CoV-2	- Nucleoprotein - Nucleocapsid protein - Protein N	-
*6YI3*	(NMR)	- SARS-CoV-2	- Nucleoprotein - Nucleocapsid protein - Protein N	-
*6VYO*	1.7	- SARS-CoV-2	- Nucleoprotein - Nucleocapsid protein - Protein N	-
*6WJI*	2.05	- SARS-CoV-2	- Nucleocapsid protein - Nucleoprotein - Protein N	-
*7C22*	2.00	- SARS-CoV-2	- Nucleoprotein - Nucleocapsid protein - Protein N	[124]
*6ZCO*	1.36	- SARS-CoV-2	- Nucleoprotein - Nucleocapsid protein - Protein N	-

**Table 6:** Vaccines available against SARS-CoV-2 infection.

Vaccine	Classification	Efficiency	Required Dose	Postvaccination Symptom	Vaccine Producer
Moderna mRNA1273	mRNA vaccine	94%	2 doses/3 weeks apart	Local and systemic reaction	Moderna, and National institute of allergy and infectious diseases, Cambridge, MA, USA
Pfizer-BioNTech (BNT162B1)	mRNA vaccine	95%	2 doses/3 weeks apart	Pain, redness, joint pain, muscle pain, paroxysmal ventricular	Pfizer (New York, NY, USA), and BioNTech, Mainz, Germany
Covaxin (BBV152)	Inactivated virus vaccine	81%	2 doses/4 weeks apart	Pain at site of injection	Bharat Biotech, Genome Valley, Hyderabad, India
Sinopharm (BBIBPCorV)	Inactivated virus vaccine	79%	2 doses/3 weeks apart	Mild cellulitis	Beijing Bio-Institute, Beijing, China
CanSino (Ad5-nCoV)	Vector vaccine	66%	1 dose	Pain at site of injection	CanSino Biologics, Tianjin, China
AstraZeneca (ChAdOx1/ AZD1222)	Vector vaccine	70%	2 doses/12 weeks apart	A pathogenic PF4-dependent syndrome may develop	AstraZeneca, Oxford, England
Jansseen (Ad26COVS1)	Vector vaccine	73%	2 doses/3 weeks apart	Irritation	Janssen Pharmaceutical Companies, Leiden, The Netherlands
SputnikV (Gram Covid Vac)	Vector vaccine	91%	2 doses/3 weeks apart	Flu-like illness, headache, asthenia, renal colic, deep vein thrombosis	Gamaleya Research Institute, Moscow, Russia
SinoVac (CoronaVac)	Virus vaccine inactivated with aluminum hydroxide	50%	2 doses/2 weeks apart	Pain, fever	Wuhan Institute, and Sinovac Biotech, Beijing, China
Novavax (NVXCoV2373)	Virus resemble vaccine particles (adjuvanted recombinant protein nanoparticle)	96%	2 doses/3 weeks apart	Fatigue, headache, pain	Novavax Biotechnology company, Gaithersburg, MD, USA

**Table 7:** Selected antiviral plants targeting coronaviruses (CoVs).

Medicinal Plants	Antiviral Compound(s)	Virus	Structure	Reference
Umbelliferae	Glycycoumarin	Inhibit SARS-CoV-2 3CLpro by hydrogen bonding interactions	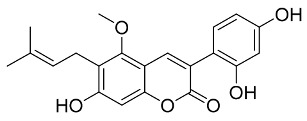	[267,277]
*Torreya nucifera*	Amentoflavone	Inhibit SARS-CoV-2 3CLpro by blocking the S-protein and ACE2 interaction	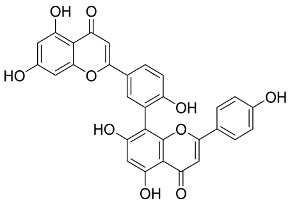	[51,278]
*Rheum officinale* Baill	Emodin	Inhibit SARS-CoV by blocking the S protein and ACE2 interaction	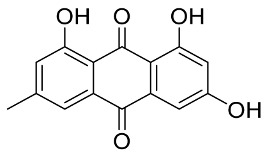	[279]
*Broussonetia papyrifera*	Broussochalcone B	CoV cysteine proteases inhibitor	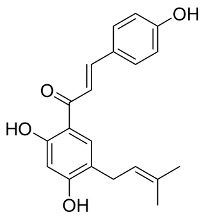	[280]
*Aglaia perviridis*	Myricetin	Inhibit the SARS-CoV helicase	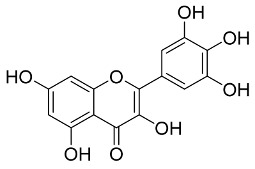	[281]
*Cinnamomi cortex*	Procyanidin A2	wtSARS-CoV and SARS-CoVS pseudovirus	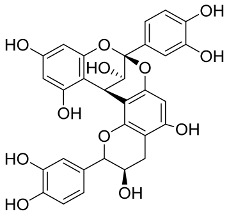	[263]
*Paulownia tomentosa*	Tomentin C3	Inhibit SARS-CoV	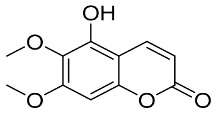	[282]
*Camellia sinensis*	Catechin gallate	Inhibit SARS-CoV N protein	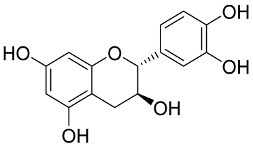	[283]
*Psoralea corylifolia*	Bavachinin	Inhibit SARS-CoV PLpro	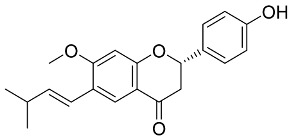	[284]
*Panax ginseng*	Ginsenoside-Rb1	Inhibit SARS-CoV replication	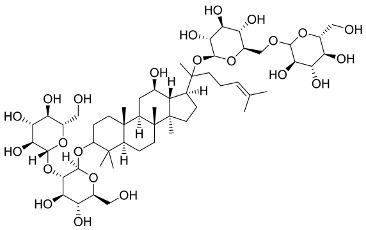	[285]
*Artemisia annua*	Arteannuin B	Inhibit SARS-CoV-2	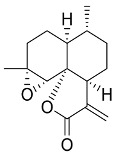	[253]
*Scutellaria baicalensis*	Baicalin	Inhibit SARS-CoV-2 by blocking the interaction with ACE2	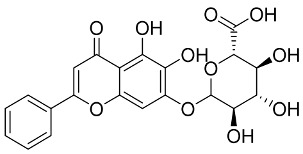	[286]
*Origanum vulgare*	Carvacrol	Inhibit SARS-CoV-2 by blocking the interaction with ACE2	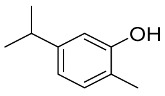	[50]
*Cymbopogonwinterianus*, *Geranium*	Citronellol	Inhibit SARS-CoV-2 proteins	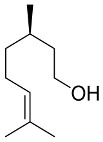	[287]
EGYVIR	Curcumin-piperine	Inhibit SARS-CoV-2	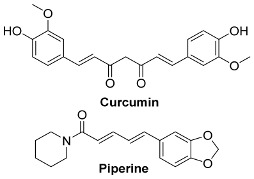	[288]
*Essential oils*	Geraniol	Inhibit SARS-CoV-2 by blocking the interaction with ACE2	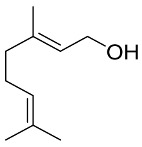	[50]
Bile acids	Glyco-ursodeoxycholic acid	Inhibit SARS-CoV-2 main protease	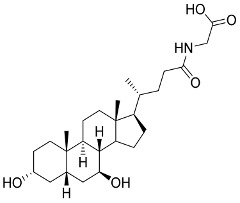	[289]
*Rhodiola rosea*	Herbacetin	Inhibit SARS-CoV-2 main protease	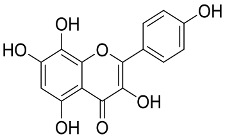	[290]
*Essential oils*	Limonene	Inhibit SARS-CoV-2 proteins	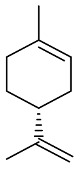	[287]
*Essential oils*	Linalool	Inhibit SARS-CoV-2 proteins	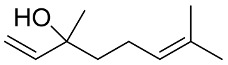	[287]
*Citrus oils*	Neryl acetate	Inhibit SARS-CoV-2 proteins	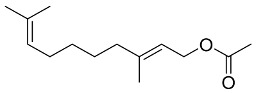	[287]
*Olea europaea*	Oleanolic acid	Inhibit SARS-CoV-2 main protease	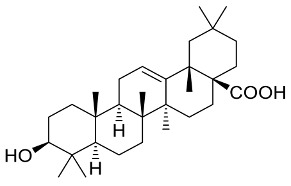	[289]
*Cirsium setidens*	Pectolinarin	Inhibit SARS-CoV-2 main protease	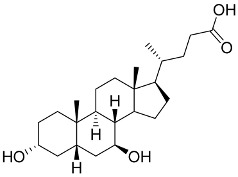	[290]
Bile Acids	Ursodeoxycholic acid	Inhibit SARS-CoV-2 main protease	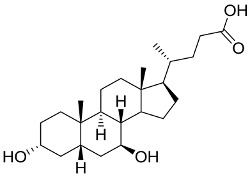	[289]
Bile Acids	Ursolic acid	Inhibit SARS-CoV-2 main protease	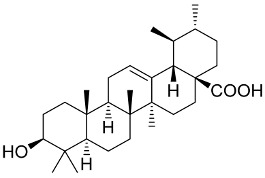	[289]

**Table 8:** Natural compounds under clinical investigations against SARS-CoV-2.

Natural Source	Antiviral Compound(s)	Chemical Structure	Reference
*Artemisia annua*	Artemisinin	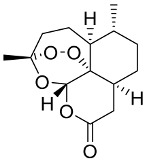	[253,304]
*Berberis vulgaris*	Berberine	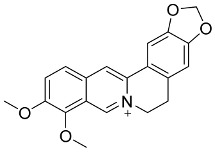	[305,306,307,308,309,310]
Bile acids	Chenodeoxycholic acid	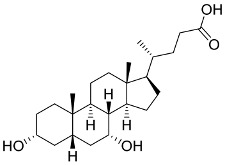	[289]
*Colchicum autumnale*	Colchicine	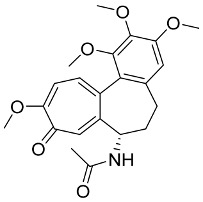	[311,312]
*Rheum officinale* and *Reynoutria* *multiflora*	Emodin	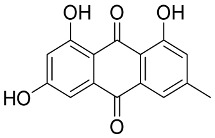	[313]
*Glycyrrhiza glabra*	Glycyrrhizin	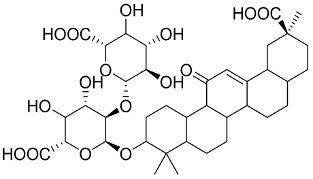	[314,315]
*Stephania tetrandra*	Hanfangchin A	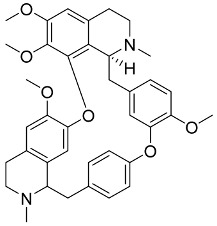	[316]
*Mammalian and Animal milk*	Lactoferrin	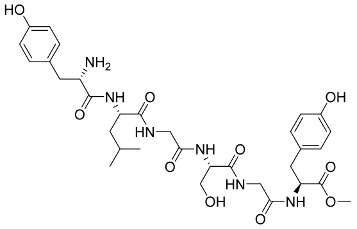	[317]
Onion, Garlic, Peppermint, Fenugreek, Origan Capers, Onions, Elderberries, Kale, Okra, Apple Peels, Aronia Berries, Cranberries, Asparagus, Goji Berries	Quercetin	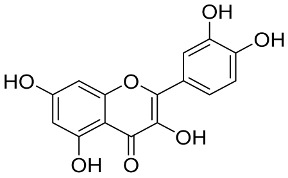	[280,318,319,320]
Red wine, Red grape juice, Peanuts, Fresh grapes, Pistachios, Peanut butter, Cocoa powder, Dark chocolate, Milk chocolate, Strawberries, Jackfruit skin, Blueberries, Bilberries, Red currants, Cranberries, Lingonberries, Mulberries	Resveratrol	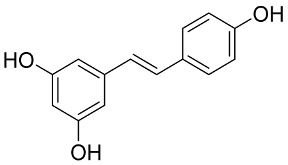	[321,322]
Citrus Fruit, Tropical Fruit, Peppers, Cruciferous Vegetables, Leafy Vegetables	Vitamin C	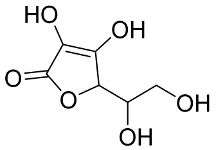	[323,324,325,326,327,328,329,330]
Human and Jay Jasmine plant	Vitamin D	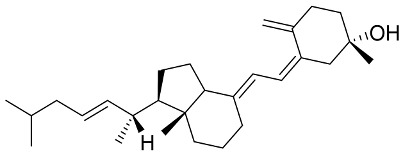	[331]

## Supplementary Materials

The following are available online at https://www.mdpi.com/article/10.3390/pharmaceutics13111759/s1, Table S1: RT-PCR primers and probes for detecting SARS-CoV-2 in different institutes, Table S2: Comparison of serological tests for COVID-19 detection, Table S3: Some of Basidiomycetes fungi and their immunological bioactive materials, and Table S4: Antivirals derived from marine algae.
