# Supplementary material for: A Comprehensive Review about the Molecular Structure of Severe Acute Respiratory Syndrome Coronavirus 2 (SARS-CoV-2): Insights into Natural Products against COVID-19
Source: Pharmaceutics. 2021 Oct 21;13(11):1759. doi: 10.3390/pharmaceutics13111759 (PMC8624722; doi:10.3390/pharmaceutics13111759)
Supplement: Supplementary file 1 [file pharmaceutics-13-01759-s001.zip › pharmaceutics-1391319 - supple.pdf]

pharmaceutics

Supplementary Information: A Comprehensive Review about the Molecular Structure of Severe Acute Respiratory Syndrome Coronavirus 2 (SARS-CoV-2): Insights into Natural Products against COVID-19

**Essa M. Saied *, Yousra A. El-Maradny, Alaa A. Osman, Amira M. G. Darwish, Hebatallah H. Abo Nahas, Gniewko Niedbała, Magdalena Piekutowska, Mohamed A. Abdel-Rahman, Bassem A. Balbool and
Ahmed M. Abdel-Azeem ***

Table S1. RT-PCR primers and probes for detecting SARS-CoV-2 in different institutes.

Institute	Gene	Probe (5'-3')	Forward primer (5'-3')	Reverse primer (5'-3')	Ref.
China CDC	ORF1ab	FAM-CCGTCTGCGGTATGTGGA AAGGTTATGG-BHQ1	CCCTGTGGGTTTTACACTTAA	ACGATTGTGCATCAGCTGA	[1]
	N	FAM-TTGCTGCTGCTTGACAGA TT-TAMRA	GGGGAACTTCTCCTGCTAGAAT	CAGACATTTTGCTCTCAAGCTG	
US CDC	N1 target	FAM-ACCCCGCATTAC GTT TGGTGGACC-BHQ	GAC CCC AAA ATC AGC GAA AT	TCT GGT TAC TGC CAG TTG AAT CTG	[2]
	N2 target	FAM-ACAATTTGCCCCCAGCGC TTCAG-BHQ1	TTA CAA ACA TTG GCC GCA AA	GCG CGA CAT TCC GAA′	
	N3 target	FAM-AYCACATTGGCACCCGCA ATCCTG-BHQ1	GC CTT GAA TAC ACC AAA A	TGT AGC ACG ATT GCA TTG	
FrancePasteur Institute	RdRp1 target	HEX-AGATGTCTTGTGCTGCCG GTA-BHQ1	ATGAGCTTAGTCCTGTTG	CTCCCTTTGTTGTGTTGT	[3]
	RdRp2 target	FAM-TCATACAAACCACGCCAG G-BHQ1	GGT TCG	CTG GG	
Japan National Institute of Infectious Disease	N	FAM-ATGTCGCGCATTGGCATG GA-BHQ	AAATTTTGGGGACCAGGAA	TGGCAGCTGTGTAGGTCAAC	[4]
Germany Charité	RdRp	FAM-CAGGTGGAACCTCATCAG GAGATGC-BBQ	GTGARATGGTCATGTGT G	CARATGTTAAASACACTATTAGCATA	[5]
	E	FAM-ACACTAGCCATCCTTACTGCGCTTCG-BBQ	ACA TAATAGCGT	ATATTGCAGCAGTACGCACACA	
Thailand National Institute of Health	N	FAM-CAACTGGCAGTAACCA-BQH1	GGTGGACCCTCAGAT	ACTGCGTTCTCCATT	[6]
Hong Kong University	ORF1b sp14	FAM-TAGTTGTGATGCWATCATGACTAG-TAMRA	TGGGGYTTTACRGGTAACCT	AACRCGCTTAACAAAGCAC	[7]
	N	FAM-GCAAATTGTGCAATTTGCGG-TAMRA	TAATCAGACAAGGAACTGATTA	CGAAGGTGTGACTTCCATG	

Table S2. Comparison of serological tests for COVID-19 detection.

Assay types	Detected antibodies	Specificity	Sensitivity	Ref.
Euroimmun (ELISA)	IgA	86.10%	83.65%	[8]
	IgG	98.60%	61.70%	
Maglumi™ (CLIA)	IgM	100.00%	58.70%	[8]
	IgG	100.00%	53.20%	
Alltest (LFA)	IgM	100.00%	28.90%	[9]
	IgG	100.00%	60.00%	
OrientGene (LFA)	IgM	95.10%	72.50%	[10]
	IgG	93.20%	68.00%	
VivaDiag (LFA)	IgM	100.00%	65.40%	[10]
	IgG	99.00%	62.80%	
StrongStep (LFA)	IgM	99.00%	32.00%	[10]
	IgG	99.00%	64.70%	
Dynamiker (LFA)	IgM	95.10%	69.30%	[10]
	IgG	99.00%	61.40%	
Multi-G (LFA)	IgM	91.30%	43.80%	[10]
	IgG	97.10%	64.70%	

Table S3. **Some of Basidiomycetes fungi and their immunological bioactive materials.**

Category	Source	Bioactive Agent	Immune Effects	Ref.
Lectins	*Volvariella volvacea*	Concanavalin A	Activating T lymphocytes	[11]
	Clitocybe nebularis	Ricin-B-like lectin	Stimulating dendritic cells (DCs) and cytokines	[12]
Fungal immunomodulatory proteins (FIPs)	*Flammulina velutipes*	FIP-fve	Stimulating lymphocyte mitogenesis, enhancing transcription of IL-2, IFN-, and TNF- α	[13]
	Ganoderma atrum	Fip-gat	Inducing apoptosis via autophagy	
	Ganoderma tsugae	Fip-gts	Inducing apoptosis via autophagy	[14]
	Ganoderma sinensis	FIP- gsi	Cytokines regulation (IL-2, IL-3, IL-4, IFN γ, TNF- α)	[15]
	Lentinus tigrinus	Fip-lti1, Fip-lti2	Cytokines regulation (TNF- α, IL-1 β, and IL-6)	[16]
	Ganoderma lucidum, Flammulina velutipes, Volvariella volvacea	FIP-SJ75	Activating macrophage M1 polarization and starting pro-inflammatory response	[17]
	Volvariella volvacea	Fip-vvo	Lymphocytes activator, cytokine regulation	[18]
	Ganoderma lucidum	Ling Zhi-8 (Lz-8)	T cell and macrophages activator, cytokine regulation	[19]
Polysaccharides	*Agaricus bisporus, Agaricus brasiliensis, Ganoderma lucidum*	Glucans	Inducing synthesis of IFN- γ	[20]
	Grifola frondosa	Glucans	Activating macrophages, NK cells, lymphokines and cytokines	[21]
	Morchella esculenta, Morchella conica	Galactomannan	Activating macrophages and cytokines	[22]
	Grifola frondosa	Grifolan	Activating macrophages and lymphokines	[23]
	Lentinus edodes	Lentinan	T-cell-oriented adjuvant	[23]
	Ganoderma lucidum	PS-G	Activating macrophages and T lymphocytes	[24]
	Schizophyllum commune	Schizophyllan	Activating T cell, increasing interleukin and TNF- α production	[25]

Terpenoids	*Ganoderma applanatum*	Exobiopolymers	Activating NK cell	[26]
	Ganoderma lucidum	Ganolucidoid A and B	NO production, anti-inflammatory activities	[11]
	Hypholoma fasciculare	Lanostane	NO production, anti-inflammatory activities	[27]

Table S4. Antivirals derived from marine algae after [28].

Compound	Source	Virus	Ref.
Calcium spirulan	*Arthrospira platensis* (Cynobacteria)	HSV-1 replication; Measles replication; Mumps replication; Influenza replication; Polio replication; Coxsackie replication; HIV-1 replication; HCMV replication; Selectively inhibition of penetration into host cells	[29]
Cyanovirin-N	*Nostoc ellipsosporum* (Cynobacteria)	HIV-1 and HIV-2 replication and fusion	[30]
Fucoidan	*Fucus vesiculosus* (Brown seaweed)	HSV-1 and HSV-2; HCMV; VSV; Sinbis virus; HIV-1 RT	[31]
Galactan Sulfate	*Aghardhiella tenera* (Red seaweed)	HIV-1 and HIV-1 CPE and syncytia formation; HIV-1 binding to host cells; Binding of anti-gpl20 mAb to HIV-1 gpl20; Other enveloped viruses (herpes viruses, togaviruses, arenaviruses, etc.)	[32]
Griffithsin	*Griffithsia* spp (Red algae)	HIV-1 glycoproteins (e.g., gpl20, gp41 and gpl60)	[33]
Sea algal extract	*Schizymenia pacifca* (Red algae)	HIV-RT and RMLV-RT	[34]
Naviculan	*Navicula directa* (Diatom)	HSV-1 and HSV-2 adhesion, penetration and replication	[35]

Herpes simplex virus (HSV), human immunodeficiency (HIV), human cytomegalovirus (HCMV), vesicular stomatitis (VSV) and rauscher murine leukemia virus (RMLV).

References

1. China-CDC Specific primers and probes for detection 2019 novel coronavirus 2020, 1–80.
2. CDC Real-time RT-PCR panel for detection 2019-Novel Coronavirus.
3. Pasteur institute Real-time RT-PCR assays for the detection of SARS-CoV-2 Available online: https://www.who.int/docs/default-source/coronaviruse/real-time-rt-pcr-assays-for-the-detection-of-sars-cov-2-institutpasteurparis. (accessed on Mar 2, 2021).
4. Naganori, N.; Shirato, K. Detection of Second Case of 2019-nCoV Infection in Japan; Department of Virology III, National Institute of Infectious Diseases.
5. Corman, V.M.; Landt, O.; Kaiser, M.; Molenkamp, R.; Meijer, A. Detection of 2019 novel coronavirus (2019-nCoV) by real-time RT-PCR. *Eurosurveillance* **2020**, *25*, 3.
6. Thailand National Institute of Health Department of Medical Sciences, Ministry of Public Health, T. Diagnostic Detection of Novel Coronavirus 2019 by Real Time RT-PCR.
7. Hong Kong University Detection of 2019 Novel Coronavirus (2019-nCoV) in Suspected Human Cases by RT-PCR.
8. Montesinos, I.; Gruson, D.; Kabamba, B.; Dahma, H.; Van den Wijngaert, S.; Reza, S.; Carbone, V.; Vandenberg, O.; Gulbis, B.; Wolff, F.; et al. Evaluation of two automated and three rapid lateral flow immunoassays for the detection of anti-SARS-CoV-2 antibodies. *J. Clin. Virol.* **2020**, *128*, 104413, doi:10.1016/j.jcv.2020.104413.
9. Pérez-García, F.; Pérez-Tanoira, R.; Romanyk, J.; Arroyo, T.; Gómez-Herruz, P.; Cuadros-González, J. Alltest rapid lateral flow immunoassays is reliable in diagnosing SARS-CoV-2 infection from 14 days after symptom onset: A prospective single-center study. *J. Clin. Virol.* **2020**, *129*, 104473.
10. Van Elslande, J.; Houben, E.; Depypere, M.; Brackenier, A.; Desmet, S.; André, E.; Van Ranst, M.; Lagrou, K.; Vermeersch, P. Diagnostic performance of seven rapid IgG/IgM antibody tests and the Euroimmun IgA/IgG ELISA in COVID-19 patients. *Clin. Microbiol. Infect.* **2020**, *26*, 1082–1087, doi:10.1016/j.cmi.2020.05.023.
11. Sze, S.C.W.; Ho, J.C.K.; Liu, W.K. Volvariella volvacea lectin activates mouse T lymphocytes by a calcium dependent pathway. *J. Cell. Biochem.* **2004**, *92*, 1193–1202, doi:10.1002/jcb.20153.
12. Svajger, U.; Pohleven, J.; Kos, J.; Strukelj, B.; Jeras, M. CNL, a ricin B-like lectin from mushroom Clitocybe nebularis, induces maturation and activation of dendritic cells via the toll-like receptor 4 pathway. *Immunology* **2011**, *134*, 409–418, doi:10.1111/j.1365-2567.2011.03500.x.
13. Chang, Y.C.; Chow, Y.H.; Sun, H.; Liu, Y.F.; Lee, Y.T.; Lue, K.H.; Ko, J.L. Alleviation of respiratory syncytial virus replication and inflammation by fungal immunomodulatory protein FIP-fve from Flammulina velutipes. *Antiviral Res.* **2014**, *110*, 124–131.
14. Li, J.R.; Cheng, C.L.; Yang, W.J.; Yang, C.R.; Ou, Y.C.; Wu, M.J.; Ko, J.L. FIP-gts potentiate autophagic cell death against cisplatin-resistant urothelial cancer cells. *Anticancer Res.* **2014**, *34*, 2973–2984.
15. Gao, Y.; Wáng, Y.; Wu, Y.; Chen, H.; Yang, R.; Bao, D. Protective function of novel fungal immunomodulatory proteins Fip-lti1 and Fip-lti2 from Lentinus tigrinus in concanavalin A induced liver oxidative injury. *Oxid. Med. Cell. Longev.* **2019**, 1–15.
16. Li, S.Y.; Shi, L.J.; Ding, Y.; Nie, Y.; Tang, X.M. Identification and functional characterization of a novel fungal immunomodulatory protein from Postia placenta. *Food Chem. Toxicol.* **2015**, *78*, 64–70, doi:10.1016/j.fct.2015.01.013.

17. Brown, G.D.; Herre, J.; Williams, D.L.; Willment, J.A.; Marshall, A.; Gordon, S. Dectin-1 mediates the biological effects of beta-glucans. *J. Exp. Med.* **2003**, *197*, 1119–1124, doi:DOI: 10.1084/jem.20021890.
18. Hsin, I.L.; Ou, C.C.; Wu, M.F.; Jan, M.S.; Hsiao, Y.M.; Lin, C.H.; Ko, J.L. GMI, an immunomodulatory protein from ganoderma microsporum, potentiates cisplatin-induced apoptosis via autophagy in lung cancer cells. *Mol. Pharm.* **2015**, *12*, 1534–1543, doi:10.1021/mp500840z.
19. Yeh, C.-H.; Chen, H.-C.; Yang, J.-J.; Chuang, W.-I.; Sheu, F. Polysaccharides PS-G and protein LZ-8 from Reishi (Ganoderma lucidum) exhibit diverse functions in regulating murine macrophages and T lymphocytes. *J. Agric. Food Chem.* **2010**, *58*, 8535–8544, doi:10.1021/jf100914m.
20. Kozarski, M.; Klaus, A.; Niksic, M.; Jakovljevic, D.; Helsper, J.P.F.G.; Van Griensven, L.J.L.D. Antioxidative and immunomodulating activities of polysaccharide extracts of the medicinal mushrooms Agaricus bisporus, Agaricus brasiliensis, Ganoderma lucidum and Phellinus linteus. *Food Chem.* **2011**, *129*, 1667–1675, doi:10.1016/j.foodchem.2011.06.029.
21. Seo, Y.-R.; Patel, D.K.; Shin, W.-C.; Sim, W.-S.; Lee, O.-H.; Lim, K.-T. Structural Elucidation and Immune-Enhancing Effects of Novel Polysaccharide from Grifola frondosa. *Biomed Res. Int.* **2019**, *2019*, 7528609, doi:10.1155/2019/7528609.
22. Su, C.A.; Xu, X.Y.; Liu, D.Y.; Wu, M.; Zeng, F.Q.; Zeng, M.Y.; Wei, W.; Jiang, N.; Luo, X. Isolation and characterization of exopolysaccharide with immunomodulatory activity from fermentation broth of Morchella conica. *DARU, J. Pharm. Sci.* **2013**, *21*, 2–7, doi:10.1186/2008-2231-21-5.
23. Murata, Y.; Shimamura, T.; Tagami, T.; Takatsuki, F.; Hamuro, J. The skewing to Th1 induced by lentinan is directed through the distinctive cytokine production by macrophages with elevated intracellular glutathione content. *Int. Immunopharmacol.* **2002**, *2*, 673–689, doi:10.1016/S1567-5769(01)00212-0.
24. Xu, H.; Kong, Y.Y.; Chen, X.; Guo, M.Y.; Bai, X.H.; Lu, Y.J.; Li, W.; Zhou, X.W. Recombinant FIP-gat, a Fungal Immunomodulatory Protein from Ganoderma atrum, Induces Growth Inhibition and Cell Death in Breast Cancer Cells. *J. Agric. Food Chem.* **2016**, *64*, 2690–2698, doi:10.1021/acs.jafc.6b00539.
25. Hobbs, C.R. The chemistry, nutritional value, immunopharmacology, and safety of the traditional food of medicinal split-gill fugus Schizophyllum commune Fr.:Fr. (Schizophyllaceae). A literature review. *Int. J. Med. Mushrooms* **2005**, *7*, 127–139, doi:10.1615/IntJMedMushr.v7.i12.130.
26. Li, Q.Z.; Zheng, Y.Z.; Zhou, X.W. Fungal immunomodulatory proteins: characteristic, potential antitumor activities and their molecular mechanisms. *Drug Discov. Today* **2019**, *24*, 307–314, doi:10.1016/j.drudis.2018.09.014.
27. Kim, K.H.; Moon, E.; Choi, S.U.; Kim, S.Y.; Lee, K.R. Lanostane triterpenoids from the mushroom Naematoloma fasciculare. *J. Nat. Prod.* **2013**, *76*, 845–851, doi:10.1021/np300801x.
28. Yasuhara-Bell, J.; Lu, Y. Marine compounds and their antiviral activities. *Antiviral Res.* **2010**, *86*, 231–240, doi:10.1016/j.antiviral.2010.03.009.
29. Hayashi, T.; Hayashi, K.; Maeda, M.; Kojima, I. Calcium spirulan, an inhibitor of enveloped virus replication, from a blue-green alga Spirulina platensis. *J. Nat. Prod.* **1996**, *59*, 83–87, doi:10.1021/np960017o.
30. Dey, B.; Lerner, D.L.; Lusso, P.; Boyd, M.R.; Elder, J.H.; Berger, E.A. Multiple Antiviral Activities of Cyanovirin-N: Blocking of Human Immunodeficiency Virus Type 1 gp120 Interaction with CD4 and Coreceptor and Inhibition of Diverse Enveloped Viruses. *J. Virol.* **2000**, *74*, 4562–4569, doi:10.1128/jvi.74.10.4562-4569.2000.
31. Pozharitskaya, O.; Obluchinskaya, E.; Shikov, A. Mechanisms of Bioactivities of Fucoidan from the Brown Seaweed Fucus vesiculosus L. of the Barents Sea. *Mar. Drugs* **2020**, *18*, 275.

32. Witvrouw, M.; Este, J.A.; Mateu, M.Q.; Reymen, D.; Andrei, G.; Snoeck, R.; Ikeda, S.; Pauwels, R.; Bianchini, N.V.; Desmyter, J. Activity of a sulfated polysaccharide extracted from the red seaweed Aghardhiella tenera against human immunodeficiency virus and other enveloped viruses. *Antivir. Chem. Chemother.* **1994**, *5*, 297–303.
33. Mori, T.; O'Keefe, B.R.; Sowder, R.C.; Bringans, S.; Gardella, R.; Berg, S.; Cochran, P.; Turpin, J.A.; Buckheit, R.W.; McMahon, J.B.; et al. Isolation and characterization of Griffithsin, a novel HIV-inactivating protein, from the red alga Griffithsia sp. *J. Biol. Chem.* **2005**, *280*, 9345–9353, doi:10.1074/jbc.M411122200.
34. Nakashima, H.; Kido, Y.; Kobayashi, N.; Motoki, Y.; Neushul, M.; Yamamoto, N. Purification and characterization of an avian myeloblastosis and human immunodeficiency virus reverse transcriptase inhibitor, sulfated polysaccharides extracted from sea algae. *Antimicrob. Agents Chemother.* **1987**, *31*, 1524–1528, doi:10.1128/AAC.31.10.1524.
35. Lee, J.B.; Hayashi, K.; Hirata, M.; Kuroda, E.; Suzuki, E.; Kubo, Y.; Hayashi, T. Antiviral sulfated polysaccharide from Navicula directa, a diatom collected from deep-sea water in Toyama Bay. *Biol. Pharm. Bull.* **2006**, *29*, 2135–2139, doi:10.1248/bpb.29.2135.
